# Diet and life history reduce interspecific and intraspecific competition among three sympatric Arctic cephalopods

**DOI:** 10.1038/s41598-020-78645-z

**Published:** 2020-12-09

**Authors:** Alexey V. Golikov, Filipe R. Ceia, Rushan M. Sabirov, Georgii A. Batalin, Martin E. Blicher, Bulat I. Gareev, Gudmundur Gudmundsson, Lis L. Jørgensen, Gazinur Z. Mingazov, Denis V. Zakharov, José C. Xavier

**Affiliations:** 1 Department of Zoology, Kazan Federal University, 420008 Kazan, Russia; 2 Department of Life Sciences, Marine and Environmental Sciences Centre, University of Coimbra, 3000-456 Coimbra, Portugal; 3 Laboratory of Isotopic and Elemental Analysis, Kazan Federal University, 420111 Kazan, Russia; 4 Greenland Climate Research Centre, Greenland Institute of Natural Resources, 3900 Nuuk, Greenland; 5 Collections and Systematics Department, Icelandic Institute of Natural History, 210 Gardabaer, Iceland; 6 Tromsø Branch, Institute of Marine Research, 9294 Tromsø, Norway; 7 Laboratory of Hydrobiology, Polar Branch of All-Russian Research Institute of Fisheries and Oceanography, 183038 Murmansk, Russia; 8 Laboratory of Zoobenthos, Murmansk Marine Biological Institute, 183010 Murmansk, Russia; 9 British Antarctic Survey, Natural Environment Research Council, Cambridge, CB3 0ET UK

**Keywords:** Stable isotope analysis, Zoology, Ecology

## Abstract

Trophic niche and diet comparisons among closely sympatric marine species are important to understand complex food webs, particularly in regions most affected by climate change. Using stable isotope analyses, all ontogenetic stages of three sympatric species of Arctic cephalopods (genus *Rossia*) were studied to assess inter- and intraspecific competition with niche and diet overlap and partitioning in West Greenland and the Barents Sea. Seven traits related to resource and habitat utilization were identified in *Rossia*: no trait was shared by all three species. High boreal *R. megaptera* and Arctic endemic *R. moelleri* shared three traits with each other, while both *R. megaptera* and *R. moelleri* shared only two unique traits each with widespread boreal-Arctic *R. palpebrosa*. Thus all traits formed fully uncrossing pattern with each species having unique strategy of resource and habitat utilization. Predicted climate changes in the Arctic would have an impact on competition among *Rossia* with one potential ‘winner’ (*R. megaptera* in the Barents Sea) but no potential ‘losers’.

## Introduction

Co-occurrence (sympatry) among species with high degree of ecological similarity leads to interspecific competition if the shared resources are limited^1–3^, especially in close-related species^4,5^. Intraspecific competition occurs within species, e.g. different ontogenetic stages and sexes^5,6^. This suggests ecological niches (which are multivariate spaces influenced by what organisms consume and the habitat in which they live, after^2^) do not completely overlap between different species, and thus the degree of niche similarity can reflect the potential competition among species^6,7^. However, the ‘[niche] overlap is only a necessary but not a sufficient condition for exploitation competition’^8^, and it does not always lead directly to competition under natural conditions, unlike what is suggested in idealized mathematical models^6,7^.

Sympatric marine species may partition habitat and resources in many ways (reviews^9,10^), sometimes resulting in asymmetric competition, when the effect of one competitor on another is greater than vice versa^11^. Thus, niche comparison among sympatric species is important to assess the mechanisms of their coexistence^7^ and to allow a better understanding of the food web functioning.

Stable isotope analysis (SIA) has shown to be a successfully applied method in trophic ecology^12,13^. Carbon (*δ*^13^C) and nitrogen (*δ*^15^N) stable isotopes are the most frequently used: *δ*^13^C shows original sources of dietary carbon (i.e. foraging habitat) and *δ*^15^N shows stepwise enrichment with each trophic step (i.e. trophic level (TL)) of species^12,13^. Thus, these parameters reflect scenopoetic (the habitat where the species live; *δ*^13^C) and bionomic (what the species consume; *δ*^15^N) axes in multivariate Hutchinson’s space, ecological niche^2^, and thus isotopic niche based on these two stable isotopes can largely reflect trophic niche of species^14–17^, although limited and not absolutely equivalent^18^. Recent Bayesian models allow estimation of robust metrics isotopic niches and to reconstruct consumers’ diets if information on isotopic signatures of their prey sources exists (reviews^19,20^). In marine ecosystems, SIA studies addressing niche partitioning between and within species mostly focus on vertebrates, namely fishes (e.g.^21,22^), seabirds (e.g.^23,24^) and marine mammals (e.g.^25–27^). There are fewer studies evaluating the niche partitioning in invertebrates, mostly focusing on deposit-feeders, filter-feeders, opportunistic predators and grazers (e.g.^28–30^). Studies on inter- and intraspecific competition in sympatric, highly mobile and obligatory carnivorous invertebrates such as the majority of cephalopods are rare^31–33^.

Cephalopods are important in marine ecosystems as both prey and predators (reviews^34,35^), and also important commercially (review^36^). Even in the Arctic, where environmental conditions are presumably less favourable for cephalopods^37–39^, recent studies demonstrate their importance in the ecosystems^37,39–43^, and higher abundance than previously thought (cf.^38,44^). Moreover, certain cephalopods are known to be influenced by climate change in the Arctic^37,39^, one of the most affected regions in the World^45^. Indeed, such a significant environmental change influence the Arctic marine ecosystems at all levels, from plankton to top predators^46,47^ and knowledge in mid-trophic organisms, such as cephalopods, is currently needed to mitigate negative consequences of climate change in the upcoming years.

There are three species of bobtail squids (Cephalopoda: genus *Rossia*) in the Arctic and high boreal Atlantic^37–39,48^: (1) *Rossia palpebrosa* Owen, 1835: a widespread boreal-Arctic species of medium size, the widest temperature range and medium habitat depth among the three species (Table 1); (2) *Rossia megaptera* Verrill, 1881: previously regarded as a western Atlantic boreal species, however it also lives in East Greenland, Iceland, Norwegian coast and reaches the Barents Sea. It has the smallest size among the three species, the highest preferable habitat temperatures and the deepest habitat (Table 1); and (3) *Rossia moelleri* Steenstrup, 1856: an Arctic endemic species, with the largest size among the three species, inhabits the coldest and the shallowest areas (Table 1). All three species are nekto-benthic and hunt as ambush predators, attacking only live prey^36,44^. However, very little information is available about their diet^48^, apart from the most abundant species, *R. palpebrosa*, whose diet was recently studied^41^. These species are sympatric in some areas of the Arctic (Table 1), and have similar sizes and supposedly similar hunting behavior. All three species play important ecological roles in the Arctic ecosystems (e.g. *R. palpebrosa* is the most abundant nekto-benthic cephalopod in the Arctic^38,43^). However, our knowledge about these species and their relationships is still very limited. We applied SIA to a representative sample of all ontogenetic stages of three species of the genus *Rossia* from the Arctic to assess: (1) *Diet* do these species partition their niches and diets to avoid interspecific competition; (2) *Life style* do they reduce interspecific competition by having different life styles, which is not obviously seen, but can be highlighted using SIA; (3) *Ontogen*y how do they cope with intraspecific competition in ontogenetic and sexual aspects. The potential impact of climate change on competition among these species was preliminary assessed.

**Table 1:** Ranges and maximum recorded sizes in the studied species of the genus *Rossia*, and exact sampling areas and corresponding environmental parameters (temperature and depth).

Species/characteristic or parameter	*Rossia palpebrosa* Owen, 1835	*Rossia megaptera* Verrill, 1881	*Rossia moelleri* Steenstrup, 1856
Biogeographic definition	Widespread boreal-Arctic	High boreal	Arctic (endemic)
Known range (from literature)	From Ellesmere Land to East Siberian Sea, south to South Carolina and The North Sea^38,48^	From Davis Strait to Nova Scotia^38,48^	From Yukon to East Siberian Sea, south to 62° N in Greenland and to 74° N in the Barents Sea^38,48,49^
Corrections to range	No corrections	Confirmed in West Greenland up to 74° N, East Greenland, Iceland, Norwegian coast and western Barents Sea*	Relatively rare in the Barents Sea
Maximum mantle length, mm	59^a^	47^a^	76^a^
Sampling area	61° 24.49′–75° 35.73′ N	60° 26.18′–73° 55.25′ N	No samples
West Greenland	50° 03.01′–65° 40.34′ W	47° 56.23′–60° 40.91′ W	
Sampling area	65° 36.82′ N	63° 14.32′–65° 52.28′ N	No samples
East Greenland	29° 35.42′ W	31° 25.97′–40° 07.38′ W	
Sampling area	70° 30.55′–81° 14.90′ N	68° 58.33′–81° 33.40′ N	75° 30.60′–80° 45.15′ N
Barents Sea	35° 35.75′–52° 47.25′ E	25° 30.30′–40° 06.25′ E	14° 34.20′–54° 26.50′ E
Sampling area	No samples	No samples	71° 16.80′–81° 17.00′ N
Kara Sea			57° 21.00′–76° 28.20′ E
Temperature range, °C	− 1.78 to 7.40 (1.14 ± 0.06)	− 0.56 to 8.37 (3.66 ± 0.11)	− 1.20 to 2.92 (0.17 ± 0.18)
Depth range, m	48.5 to 617 (250.5 ± 3.3)	54 to 1169.5 (329.4 ± 10.0)	50 to 397 (204.9 ± 13.1)

Values of environmental parameters are minimum − maximum (mean ± SE).

*Reasons why *R. megaptera* was overlooked for a long time on such huge areas and related details are work in progress (Golikov et al. in prep.). Presence of this species in Iceland was recently published^50^.

^a^New maximum mantle length of these species, exciding previous published records (cf.^38,48,49,51^).

## Methods

### Study area and samples

Material was collected from various Arctic regions: the samples from Greenland were obtained by R/V ‘Paamiut’ (2016–2017) and F/V ‘Helga Maria’ (2019), samples from the Barents and Kara Seas were obtained by R/Vs ‘Vilnus’ (2003–2017), ‘Nansen’ (2006, 2007), ‘Smolensk’ (2007) and ‘Dalnie Zelentsy’ (2007, 2017) (Table 1). *Rossia palpebrosa* (*n* = 49), *R. megaptera* (*n* = 45) and *R. moelleri* (*n* = 39) were collected in July–August (see Table 2, Supplementary Tables S1–S4, for detailed information per species, area, sex and life-stage). All the studied species are known to grow continuously throughout their life cycle, while having highly variable size at maturity^49,51^ (Golikov et al., unpubl.). Thus, all specimens were categorized in three (*R. moelleri* in four) arbitrary ontogenetic size groups: mantle length (ML) < 21 mm (small), ML 21 to 40 mm (medium) and ML > 41 mm (large), corresponding roughly to the life-stages of immature, maturing and mature specimens, respectively. In *R. moelleri*, large specimens were categorized as ML 41 to 60 mm, and a fourth group, very large, as ML > 61 mm: they all were mature females. Eight specimens of all groups were randomly selected for SIA but all specimens were taken if less than *n* = 8 existed in any group (Tables 2, 3, Supplementary Tables S1–S4). Some of *R. palpebrosa* samples (*n* = 37) were used in recent SIA study of species’ stomach contents ^41^.

**Table 2:** Mantle length (ML), values of *δ*^13^C and *δ*^15^N and estimated trophic level (TL) in the studied species of the genus *Rossia* by stage and sex, and for pooled data.

	All	Small	Medium	Large	Very large	Females	Males
***Rossia palpebrosa***							
*n*	49	16	17	16	–	26	23
ML, mm	10 to 56 (30.7 ± 2.0)	10 to 19 (14.4 ± 0.8)	22 to 40 (30.9 ± 1.3)	41 to 56 (46.8 ± 1.1)	–	10 to 56 (31.8 ± 2.7)	10 to 56 (29.4 ± 3.0)
*δ*^13^C, ‰	− 21.6 to − 17.0 (− 19.2 ± 0.2)	− 21.6 to − 17.1 (− 19.4 ± 0.3)	− 20.9 to − 17.0 (− 19.0 ± 0.3)	− 21.1 to − 17.7 (− 19.1 ± 0.2)	–	− 21.2 to − 17.0 (− 19.0 ± 0.2)	− 21.6 to − 17.5 (− 19.4 ± 0.2)
*δ*^15^N, ‰	6.0 to 11.4 (8.7 ± 0.2)	6.4 to 10.2 (8.0 ± 0.3)	6.0 to 10.1 (8.4 ± 0.3)	8.3 to 11.4 (9.6 ± 0.2)	–	6.6 to 11.4 (8.8 ± 0.2)	6.0 to 11.2 (8.5 ± 0.3)
TL	2.7 to 4.2 (3.5 ± 0.05)	2.9 to 4.0 (3.4 ± 0.1)	2.7 to 3.9 (3.5 ± 0.1)	3.4 to 4.2 (3.8 ± 0.1)	–	2.9 to 4.2 (3.6 ± 0.1)	2.7 to 4.1 (3.5 ± 0.1)
***Rossia megaptera***							
*n*	45	15	22	8	–	26	19
ML, mm	10 to 47 (25.8 ± 1.6)	10 to 18 (13.8 ± 0.7)	21 to 39 (27.9 ± 1.1)	41 to 47 (42.8 ± 0.8)	–	10 to 47 (28.8 ± 2.4)	10 to 35 (21.7 ± 1.7)
*δ*^13^C, ‰	− 21.0 to − 16.6 (− 18.9 ± 0.2)	− 20.1 to − 16.6 (− 18.6 ± 0.2)	− 20.9 to − 16.8 (− 19.0 ± 0.2)	− 21.0 to − 17.6 (− 19.3 ± 0.5)	–	− 21.0 to − 16.6 (− 19.0 ± 0.2)	− 20.7 to − 17.3 (− 18.8 ± 0.2)
*δ*^15^N, ‰	6.1 to 10.1 (8.3 ± 0.1)	6.1 to 9.2 (8.0 ± 0.2)	6.5 to 10.1 (8.4 ± 0.2)	7.1 to 9.9 (8.8 ± 0.3)	–	6.1 to 9.9 (8.3 ± 0.2)	6.6 to 10.1 (8.3 ± 0.2)
TL	2.8 to 3.8 (3.4 ± 0.04)	2.8 to 3.7 (3.3 ± 0.1)	2.9 to 3.8 (3.4 ± 0.1)	3.1 to 3.8 (3.5 ± 0.1)	–	2.8 to 3.8 (3.4 ± 0.1)	2.9 to 3.8 (3.4 ± 0.1)
***Rossia moelleri***							
*n*	39	2	19	12	6	19	20
ML, mm	9 to 76 (40.8 ± 2.5)	9 to 12 (10.5 ± 1.5)	21 to 40 (32.1 ± 1.6)	42 to 58 (46.0 ± 1.7)	62 to 76 (68.2 ± 1.9)	9 to 76 (45.7 ± 4.4)	12 to 46 (36.2 ± 2.2)
*δ*^13^C, ‰	− 23.7 to − 19.6 (− 22.1 ± 0.2)	− 22.1 to − 19.6 (− 20.8 ± 1.3)	− 23.4 to − 20.2 (− 21.9 ± 0.2)	− 23.7 to − 20.6 (− 22.4 ± 0.2)	− 22.8 to − 21.5 (− 22.4 ± 0.2)	− 23.2 to − 19.6 (− 21.9 ± 0.3)	− 23.7 to − 20.2 (− 22.2 ± 0.2)
*δ*^15^N, ‰	6.5 to 11.3 (9.3 ± 0.2)	6.5 to 8.1 (7.3 ± 0.8)	7.1 to 10.6 (8.9 ± 0.2)	8.4 to 10.4 (9.5 ± 0.2)	9.7 to 11.3 (10.5 ± 0.2)	6.5 to 11.3 (9.3 ± 0.3)	7.6 to 10.6 (9.2 ± 0.2)
TL	3.1 to 4.2 (3.7 ± 0.05)	3.1 to 3.3 (3.2 ± 0.1)	3.1 to 4.1 (3.6 ± 0.1)	3.5 to 4.1 (3.8 ± 0.1)	3.9 to 4.2 (4.0 ± 0.1)	3.1 to 4.2 (3.7 ± 0.1)	3.2 to 4.1 (3.7 ± 0.1)

Values are minimum − maximum (mean ± SE), *n* sample size.

**Table 3:** Isotopic niche metrics (TA, SEA_c_ and SEA_b_) for the studied species of the genus *Rossia* in each studied area and for pooled data, and respective differences in niche widths (*p* value), and niche overlap.

Area/parameter	Overall			Barents Sea			West Greenland	
Group	*R. palpebrosa*	*R. megaptera*	*R. moelleri*	*R. palpebrosa*	*R. megaptera*	*R. moelleri*	*R. palpebrosa*	*R. megaptera*
*n*	49	45	39	18	12	17	30	25
TA	3.77	3.27	3.61	2.53	0.63	2.11	2.13	2.55
SEA_c_	1.07	0.91	0.92	0.99	0.35	0.93	0.68	0.87
SEA_b_	1.07 ± 0.16	0.91 ± 0.14	0.92 ± 0.15	0.99 ± 0.25	0.36 ± 0.11	0.93 ± 0.25	0.68 ± 0.13	0.86 ± 0.18
*R. palpebrosa* (*p*-value)	–	0.204	0.237	–	**0.004**	0.415	–	0.8025
*R. megaptera* (*p*-value)	0.796	–	0.524	**0.996**	–	**0.9895**	0.1975	–
*R. moelleri* (*p-*value)	0.763	0.476	–	0.585	**0.0105**	–	–	–
Overlap, *R. palpebrosa*–*R. megaptera*, %	**69.9** *R. palpebrosa*; **82.4** *R. megaptera*			32.3 *R. palpebrosa*; **91.1** *R. megaptera*			**82.2** *R. palpebrosa*; **64.6** *R. megaptera*	
Overlap, *R. palpebrosa*–*R. moelleri*, %	No overlap			21.8 *R. palpebrosa*; 23.3 *R. moelleri*			–	
Overlap, *R. megaptera*–*R. moelleri*, %	No overlap			1.4 *R. megaptera*; 0.5 *R. moelleri*			–	

SEA_b_ values are means ± SD.

Significant *p*-values and large overlap values are in bold.

Specimens were fixed in formalin onboard. Mantle length was measured, and sex and maturity stage were assessed in fixed specimens onshore. Lower beaks were taken for SIA, as they have been repeatedly used in related studies (e.g.^41,42,52–54^), and their rostrums measured (*n* = 133).

### Stable isotope analysis

Transparent areas of the beaks were removed before proceeding with SIA, as they have different isotopic concentrations biasing the outputs^55^. The beaks were dried at 60 °C and ground into a fine powder. Powder sub-samples were weighed (to the nearest 0.3 mg) with a micro-balance and sterile-packed in tin containers. The analyses were carried out at the Marine and Environmental Science Centre (MARE)—University of Coimbra (Portugal) with Flash EA 1112 series elemental analyzer coupled online via a Finnigan ConFlo II interface to a Delta VS mass spectrometer (ThermoFisher Scientific) and at the Laboratory of Isotopic and Elemental Analysis—Kazan Federal University (Russia) with Flash HT series elemental analyzer coupled online via a ConFlo IV interface to a Delta V Plus mass spectrometer (ThermoFisher Scientific). No significant differences in SIA were found between the specimens of the same species and group from the same area measured in both spectrometers (*n* = 10, *U* = 23.5, *p* = 0.31). Stable isotope values were expressed as: *δ*^13^C and *δ*^15^N = [(R_sample_/R_standard_) − 1] × 1000, where R = ^13^C/^12^C and ^15^N/^14^N, respectively. The isotope ratios were expressed in delta (*δ*) notation relative to Vienna-PDB limestone (V-PDB) for *δ*^13^C and atmospheric nitrogen (AIR) for *δ*^15^N. Replicate measurements of internal laboratory standards (acetanilide STD: Thermo Scientific PN 338 36700) in every batch (*n* = 14) indicated precision < 0.2‰ for both *δ*^13^C and *δ*^15^N values. Mean mass C:N ratio were 3.34 ± 0.03, 3.39 ± 0.03 and 3.49 ± 0.03 (mean ± SE) in *R. palpebrosa*, *R. megaptera* and *R. moelleri*, respectively, with no differences among species (*H*_2,133_ = 21.54, *p* = 0.47).

### Data analyses

Differences in *δ*^13^C and *δ*^15^N values, and TLs among species, sexes, geographic areas (i.e. West and East Greenland, the Barents and Kara Seas) and size groups (i.e. small, medium, large and very large) were assessed with a Kruskal–Wallis *H* or a Mann–Whitney *U* test^56^. A regression analysis was used to find equations fitting our data^56^. All tests were performed using *α* = 0.05.

Neither ethanol nor formalin fixation significantly affects *δ*^13^C or *δ*^15^N signatures of cephalopod beaks^57^, thus no corrections were performed due to fixation. Values of *δ*^15^N in cephalopod beaks, in contrast to *δ*^13^C values, are in average 4.8‰ lower than values from muscle tissue^52,53,57,58^. Therefore, this value was subtracted from muscle *δ*^15^N values available in the literature to enable comparison with the data reported here. However, when estimating TL, we added 4.8‰ to raw beak *δ*^15^N values, as proposed by^41,42,52,54^.

Trophic level can be estimated with fixed trophic enrichment factor (TEF), either ‘classical’ *δ*^15^N = 3.4‰^59^ or ‘Arctic’ *δ*^15^N = 3.8‰^60^, and with standard TL equation^61^, or with scaled TEF equation^62,63^, adapted for the Arctic by Linnebjerg et al.^64^. We used the latter as the most up-to-date approach. Reference values for TL = 2.0 were: *δ*^15^N = 7.92‰ in Greenland (i.e. mean value of *Calanus finmarchicus*^64^); *δ*^15^N = 7.20‰ in the Barents Sea (i.e. mean value of *C. glacialis*^65^); *δ*^15^N = 7.84‰ in the Kara Sea (i.e. mean value of *C. glacialis*; Golikov et al., unpubl.). Interpretation of TLs in the Arctic ecosystems followed recent stable isotope studies of the area^41,42,60,64–67^.

Isotopic niche widths and overlap were assessed with SIBER 2.1.4^15^ in R 3.6.3^68^. The standard ellipse area corrected for small sample sizes (SEA_c_, an ellipse that contains 40% of the data regardless of sample size) and the Layman metric of convex hull area (TA) were estimated^15–17^, and the Bayesian approximation of the standard ellipse area (SEA_b_) was adopted to compare niche width among groups^15^. Large (*n* = 12) and very large (*n* = 6) specimens of *R. moelleri* were pooled in the same group (Table 3), due to the small sample size for isotopic niches’ analyses^69^. The overlap interpretation followed Langton^70^, where overlap ranged from 0.0 to 0.29 indicating no overlap, from 0.30 to 0.60 indicating medium overlap, and from 0.61 to 1.00 indicating large overlap and the latter only taken as significant, i.e. potential competition.

Trophic levels were used instead of *δ*^15^N values (Y axis) in niche estimations. This approach improves the ecological meaning of isotopic data when comparing specimens from different areas and ecosystems due to differences in baseline *δ*^15^N values (e.g.^52,64^). This approach has been repeatedly applied to cephalopods^41,54^.

The newest Bayesian mixing model, i.e. SIMMR 0.4.1^71^ in R 3.6.3^68^ was used to assess relative contribution of prey to the diet of *Rossia*. All three species were reported to eat crustaceans and fishes in Canada^48^. Stomach content analysis showed the main prey of *R. palpebrosa* in the Barents Sea are Crustacea, Polychaeta and fishes^41^, and these taxa were used as prey group sources in our models. The models were performed for the Barents Sea and West Greenland: mean source values are detailed in Table 4. All the source values were significantly different in at least one of the isotopes (Table 4). Values and standard deviations of TEF were taken from the only experimental study showing differences between cephalopod beaks and long-time diet composition^58^: *δ*^13^C =  − 0.20 ± 0.55‰ and *δ*^15^N = 3.37 ± 0.99‰. The data fitting to selected prey source values and TEFs was checked using simulated mixing polygons^72^ in R 3.6.3^68^ (Supplementary Fig. S1). Only the fitting specimens were used in models (Table 4). Individual-based models were performed for all specimens fitting the model (Supplementary Fig. S2). Diet derived from the models was compared among species (overall models), sexes, geographic areas and size groups with *χ*^2^ and Fisher’s exact tests: although the latter is more adequate for small sample sizes, Fisher’s exact allows comparison of only two groups^56^.

**Table 4:** Values of *δ*^13^C and *δ*^15^N for the prey group sources used in Bayesian mixing model SIMMR 0.4.1, and their predicted relative contribution to the diet in the studied species of the genus *Rossia*.

Prey sources	*δ*^13^C, ‰^a^	*δ*^15^N, ‰^a^	*R. palpebrosa* Barents Sea	*R. palpebrosa* West Greenland	*R. megaptera* Barents Sea	*R. megaptera* West Greenland	*R. moelleri* Barents Sea
*n*, fitting specimens*	–	–	16	22	12	16	8
*n*, outliers*	–	–	2	8	0	9	9
**Barents Sea**							
Crustacea, *n* = 63^66,67^	− 19.79 ± 1.19	10.09 ± 1.72	44.0 ± 14.7	–	45.5 ± 15.9	–	29.1 ± 15.6
Polychaeta, *n* = 18^66,67^	− 18.21 ± 1.94	11.65 ± 1.62	22.7 ± 8.5	–	30.9 ± 10.9	–	16.0 ± 8.5
Fishes, *n* = 12^66,67^	− 20.80 ± 0.28	10.15 ± 2.31	33.3 ± 11.2	–	23.6 ± 11.1	–	54.9 ± 15.6
**West Greenland**							
Crustacea, *n* = 45^60,64,79^	− 19.35 ± 1.44	10.00 ± 1.48	–	49.8 ± 10.2	–	58.3 ± 8.8	–
Polychaeta, *n* = 6^60^	− 17.90 ± 0.55	13.20 ± 1.07	–	34.0 ± 9.0	–	23.8 ± 8.4	–
Fishes, *n* = 7^60^	− 19.67 ± 0.99	11.86 ± 2.20	–	16.2 ± 8.1	–	17.9 ± 9.0	–

Values of *δ*^13^C and *δ*^15^N and relative contributions are mean ± SD.

Significant *p*-values are in bold.

*See “Methods” section for fitting checks. Only the fitting specimens were used.

^a^Significant differences between source values (Kruskal–Wallis *H* test): Barents Sea, *δ*^13^C *H*_2,93_ = 25.63, *p* < **0.0001** (Crustacea–Polychaeta *U* = 269.5, *p* = **0.0022**; Crustacea–Fishes *U* = 142.5, *p* = **0.0020**; Polychaeta–Fishes *U* = 3.5, *p* < **0.0001**); Barents Sea, *δ*^15^N *H*_2,93_ = 10.92, *p* = **0.0043** (Crustacea–Polychaeta *U* = 280, *p* = **0.0011**; Crustacea–Fishes *U* = 329.5, *p* = 0.49; Polychaeta–Fishes *U* = 59.5, *p* = **0.0420**); West Greenland, *δ*^13^C *H*_2,58_ = 8.82, *p* = **0.0121** (Crustacea–Polychaeta *U* = 41, *p* = **0.0062**; Crustacea–Fishes *U* = 144.5, *p* = 0.74; Polychaeta–Fishes *U* = 1, *p* = **0.0034**); West Greenland, *δ*^15^N *H*_2,58_ = 15.39, *p* = **0.0005** (Crustacea–Polychaeta *U* = 10, *p* = **0.0004**; Crustacea–Fishes *U* = 15, *p* = **0.0302**; Polychaeta–Fishes *U* = 84, *p* = **0.0430**).

Statistical analyses were performed in R 3.6.3^68^ and PAST 3.25^73^. Values are presented as mean ± SE unless otherwise stated.

### Ethical approval

No ethical approval was required. Beaks were only obtained from either dead or preserved specimens. No live animals were caught specifically for this project.

## Results

The known geographic ranges were expanded for *R. megaptera* and corrected for *R. moelleri*, and new maximum body sizes were recorded for all the studied species (Table 1).

### Stable isotopic values and trophic levels

Values of *δ*^13^C and *δ*^15^N varied respectively from − 23.7 to − 16.6‰ and from 6.0 to 11.4‰ (TLs from 2.7 to 4.2) in all three species of the genus *Rossia* (Table 2). *Rossia palpebrosa* had the highest variation of all values, *R. megaptera* had the lowest variation of *δ*^15^N and TL, and *R. moelleri* had the lowest variation of *δ*^13^C (Table 2). No significant ontogenetic increase of *δ*^13^C values was found in any of the studied species (Table 2, Supplementary Table S5). Significant ontogenetic decrease of *δ*^13^C values was found in *R. megaptera* in the Barents Sea and East Greenland, and in *R. moelleri* in the Barents Sea (Table 2, Supplementary Table S5). As expected, values of *δ*^15^N and TLs showed significant ontogenetic increase in all species and areas (except for *R. megaptera* in East Greenland) (Table 2, Supplementary Table S5). The largest size group was the most different from the smallest and second-most from middle one, with no differences between the smallest and middle-sized groups (Table 2, Supplementary Tables S2–S5).

A westward significant increase of *δ*^13^C values was found in *R. palpebros*a (i.e. Barents Sea–West Greenland: *U* = 81, *p* = 0.0002) and in *R. moelleri* (i.e. Kara Sea–Barents Sea: *U* = 71, *p* = 0.0005) (Table 2, Supplementary Tables S3, S4, S6). Values of *δ*^15^N, as well as TLs, showed no geographic differences, with the exceptions of *R. megaptera* which had significantly higher TL in the Barents Sea, than in East Greenland (*U* = 14, *p* = 0.0293), and *R. moelleri* which had significantly higher TL in the Barents Sea, than in the Kara Sea (*U* = 115, *p* = 0.0429) (Table 2, Supplementary Tables S3, S4, S6). No differences between sexes were found in either *δ*^13^C or *δ*^15^N values or TLs in any species (Table 2, Supplementary Tables S3, S4, S6).

Overall (i.e. using all the specimens), *Rossia moelleri* had significantly lower *δ*^13^C values than *R. palpebrosa* and *R. megaptera* (*U* = 64, *p* < 0.0001 vs. *R. palpebrosa* and *U* = 38, *p* < 0.0001 vs. *R. megaptera*), and in all areas and ontogenetic stages (Table 2, Supplementary Tables S2–S4, S7). Values of *δ*^15^N and TLs in *R. moelleri* were significantly higher than in *R. palpebrosa* and *R. megaptera*, overall (*δ*^15^N: *U* = 673, *p* = 0.0179 vs. *R. palpebrosa* and *U* = 432, *p* < 0.0001 vs. *R. megaptera*; TL: *U* = 644, *p* = 0.0086 vs. *R. palpebrosa* and *U* = 420.5, *p* < 0.0001 vs. *R. megaptera*) and in all the studied areas (Table 2, Supplementary Tables S2–S4, S7). In terms of size groups’ comparison among species, only very large *R. moelleri* had significantly higher values than large *R. palpebrosa* and *R. megaptera* (Table 2, Supplementary Tables S2–S4, S7).

### Isotopic niches

**Figure 1:**
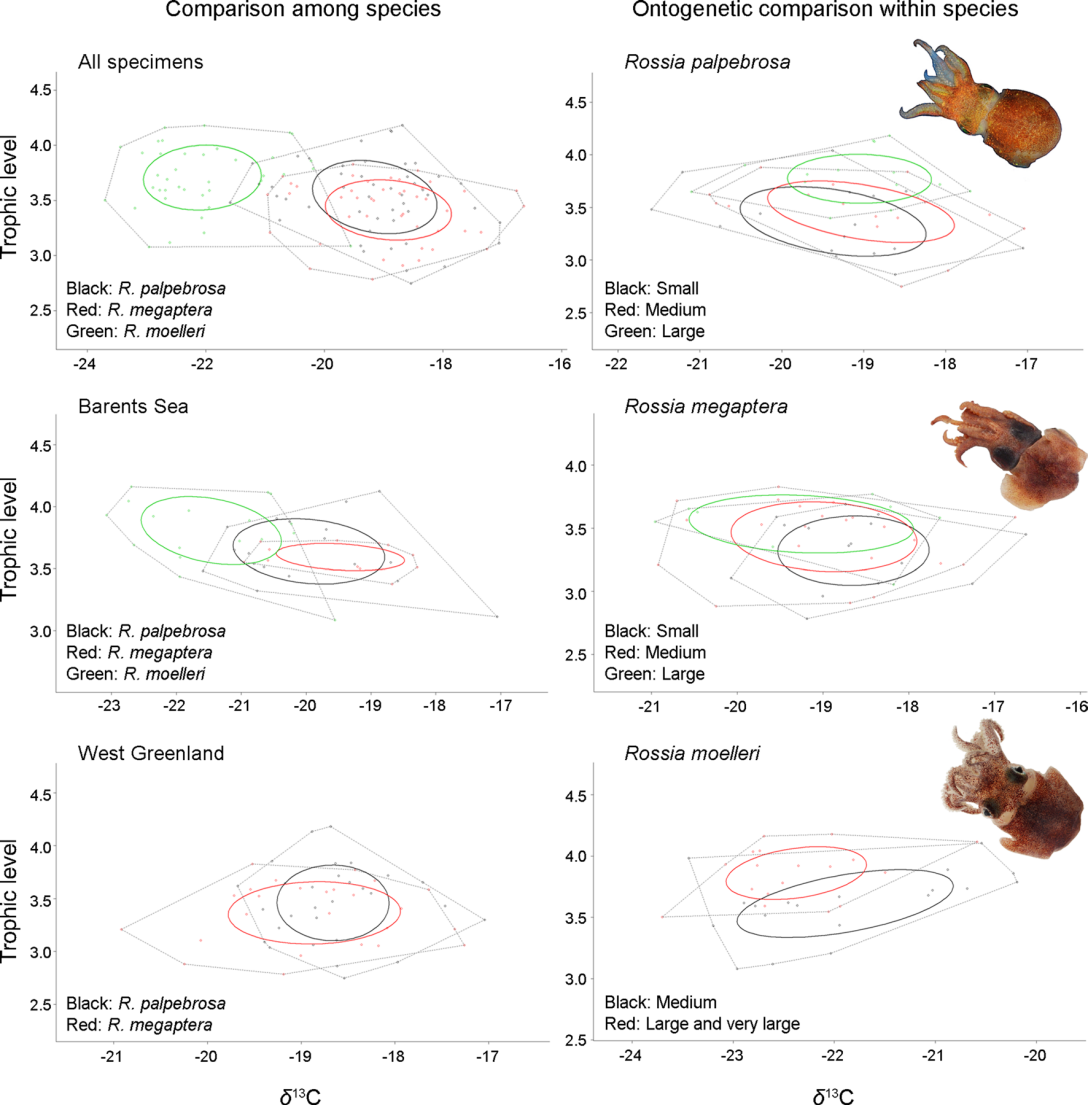
Isotopic niches of the studied species of the genus *Rossia*: comparison among species and ontogenetic comparison within species. Photo credits: Olga L. Zimina (*R. moelleri*).

No differences in niche width were found between sexes in *R. palpebrosa*; both sexes showed a large overlap (Supplementary Table S8). However, females in *R. megaptera* and *R. moelleri* had significantly wider niche than males, with males having larger overlap with females (> 95%) than vice versa (52–55%): females had medium overlap with males (Supplementary Table S8). Significant ontogenetic decrease in niche width was found in *R. moelleri*, and gradual (not significant) ontogenetic decrease and increase in *R. palpebrosa* and *R. megaptera* (Fig. 1, Supplementary Table S9). Larger size groups overlapped more with smaller ones in *R. palpebrosa* and *R. moelleri*, with the opposite pattern in *R. megaptera* (Fig. 1, Supplementary Table S9). Large overlap was found between small and medium *R. palpebrosa*, and consequently in small–medium–large *R. megaptera* (Fig. 1, Supplementary Table S9).

Differences in niche width among species were found only in the Barents Sea (Fig. 1, Table 3). In the Barents Sea, *R. megaptera* had significantly narrower niche than *R. palpebrosa* and *R. moelleri* (Fig. 1, Table 3). *Rossia moelleri* had only small overlap with *R. palpebrosa* and *R. megaptera* in the Barents Sea, and no overlap with them overall (Fig. 1, Table 3). *Rossia palpebrosa* and *R. megaptera* mostly had large overlap with each other, except for the Barents Sea, where *R. palpebrosa* had medium overlap with *R. megaptera* (Fig. 1, Table 3). *Rossia palpebrosa* overlapped more with *R. megaptera*, overall and in the Barents Sea, and the opposite in West Greenland (Fig. 1, Table 3).

### Diet models

**Figure 2:**
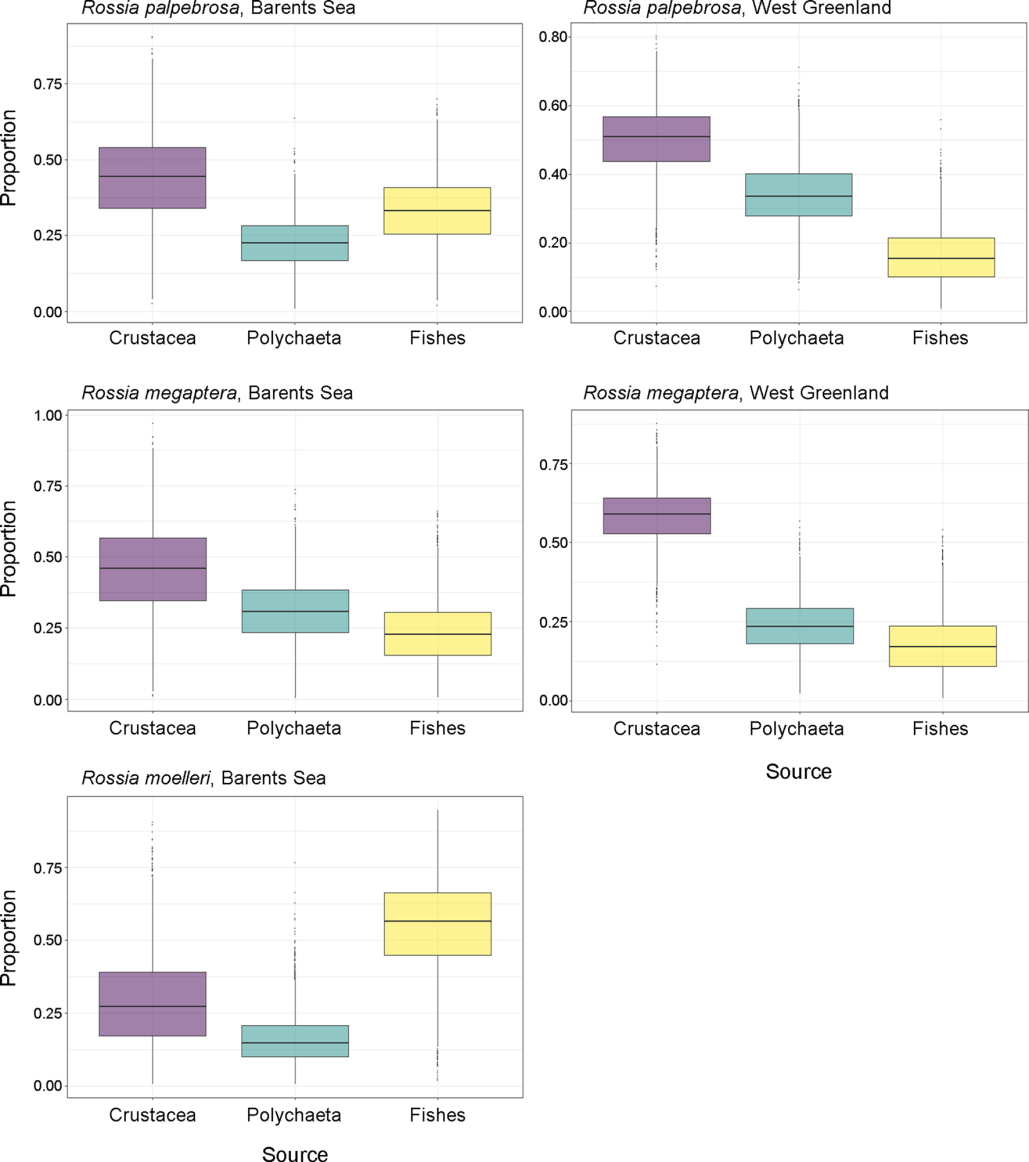
Relative contribution of prey to the diet (mean, box 25% and 75% percentiles, whiskers 5% and 95% percentiles) of the studied species of the genus *Rossia* predicted by Bayesian mixing model SIMMR 0.4.1.

The predicted diet of *R. palpebrosa* had crustaceans as the most important component in the Barents Sea (mean ± SD: 44.0 ± 14.7%) and West Greenland (49.8 ± 10.2%); crustaceans were followed by fishes (33.3 ± 11.2%) and polychaetes (22.7 ± 8.5%) in the Barents Sea, and by polychaetes (34.0 ± 9.0%) and fishes (16.2 ± 8.1%) in West Greenland (Fig. 2, Table 4). The diet of *R. palpebrosa* from the Barents Sea was significantly different from all other predicted diets, except for *R. megaptera* from the Barents Sea (Supplementary Table S10). The predicted diet of *R. megaptera* consisted of crustaceans–polychaetes–fishes in the Barents Sea and West Greenland (45.5 ± 15.9%, 30.9 ± 10.9% and 23.6 ± 11.1% and 58.3 ± 8.8%, 23.8 ± 8.4% and 17.9 ± 9.0%, respectively) (Fig. 2, Table 4). No significant differences between the areas were found in the predicted diet of this species (Supplementary Table S10). The predicted diet of *R. moelleri* had fishes as the most important component (54.9 ± 15.6%), followed by crustaceans (29.1 ± 15.6%) and polychaetes (16.0 ± 8.5%) (Fig. 2, Table 4). It was significantly different from all other predicted diets (Supplementary Table S10). Individual-based models did not demonstrate significant differences either among species, areas, sexes or size groups (Supplementary Table S11), highlighting high variation of each diet component among individuals (Supplementary Fig. S2).

## Discussion

This study assessed a long time series during which the samples were collected in the Barents and Kara Seas (2003–2017). We assume the potential biases which can possibly arise have been countered: (a) changes in *δ*^13^C values due to oceanic Suess effect were minimal (− 0.018‰^74^) and already proven negligible in Arctic fishes and marine mammals^75^; (b) to our knowledge there is a lack of long-term direct baseline variation studies in the Arctic, and the only available long-term studies for plankton and walruses *Odobenus rosmarus* showed no significant changes in *δ*^13^C and *δ*^15^N values over long time periods in high Arctic Canada^27,75^; and (c) all specimens were collected in the same years and during July–August, minimizing seasonal changes. Cephalopod beaks have recently been proven to be ‘chemical archives’ of the individual’s life^76–78^. The analysis of the whole beak can be thus a proxy of full ontogenesis of the specimen. Seasonal changes can be accessed either by analyzing different regions of the beaks synthesized during specific periods^77,78^ or by equal sample distribution throughout the year; the ‘whole-beaks approach’ applied in this study is more general, and most likely the majority of the revealed relationships are for the whole life history of the animal.

In some cases it is obvious how sympatric species decrease competition: e.g. when they demonstrate significant size, life style of habitat differences (e.g.^23,24,26,28–33^). However, the three studied species of the genus *Rossia* had largely similar body sizes, often occurred in the same trawl station, and were supposed to have similar hunting behavior, i.e. had no preliminary highlighting how they decrease competition. So, how do *Rossia* deal with potential competition? Using SIA and its applications to assess diet, life style and ontogeny, we were able to identify seven traits related to resource and habitat utilization in the three species of the genus *Rossia*: (1) *R. moelleri* had more pelagic life style, than initially supposed, while *R. megaptera* and *R. palpebrosa* had ‘typical’ life style for sepiolids; (2) *R. megaptera* and *R. moelleri* showed spatial migrations, while *R. palpebrosa* was presumably sedentary; (3) *R. megaptera* and *R. moelleri* had more pronounced sexual dimorphism in body size, and niche width in females was significantly larger than in males, suggesting asymmetrical competition, where large and very large females are in competitive advantage; (4) *R. megaptera* and *R. moelleri* showed a less varying diet between regions, than *R. palpebrosa*; (5) *R. megaptera* and *R. palpebrosa* had crustaceans as their main prey, while fishes dominated in *R. moelleri*; (6) *R. palpebrosa* and *R. moelleri* had ontogenetic decrease in isotopic niche width (common for cephalopods), while *R. megaptera* showed ontogenetic increase; and (7) *R. palpebrosa* and *R. moelleri* showed similar strategies to reduce intraspecific competition, different from *R. megaptera*: asymmetrical competition favours smaller-sized groups in the both former species and all stages are largely overlapping, while larger-sized groups are favoured in *R. megaptera*. No trait was shared by all three species, and high boreal *R. megaptera* and Arctic endemic *R. moelleri* shared three traits with each other, while both *R. megaptera* and *R. moelleri* shared only two unique traits each with widespread boreal-Arctic *R. palpebrosa*. Thus all traits formed fully uncrossing pattern with each species having unique strategy of resource and habitat utilization.

How the diet specialization and its ontogenetic changes are a means to reducing competition? These species of the genus *Rossia* belong to Arctic nekto-benthic predators’ trophic guild, which includes large shrimps and fishes. However, shrimps and fishes present a wider diet spectrum (often scavenge) and thus a wide range of both *δ*^13^C and *δ*^15^N values^60,64,66,67,75,79^. Westward significant increase of *δ*^13^C values, which is usually found in different taxa from the Arctic marine ecosystems^41,42,60,64,65,67,79^, was found in *R. palpebrosa* and *R. moelleri*, and lacked in *R. megaptera*. Significantly higher *δ*^15^N values and TLs in *R. moelleri* than in *R. palpebrosa* and *R. megaptera* suggested marked dietary differences, which were also highlighted by SIMMR: crustaceans were the most important group in diet of *R. palpebrosa* and *R. megaptera*, and fishes in *R. moelleri*. *Rossia moelleri* had the most different diet among *Rossia*, and is the only sepiolid in the world ocean whose main prey are fishes (reviews^36,44^). *Rossia palpebrosa* had more varying diet between the studied areas than *R. megaptera*.

In general, all three species had lower *δ*^15^N values and TLs than North Atlantic squids, and similar or higher than octopods, cuttlefishes and sepiolids^58,76,80–82^. Ontogenetic increase of *δ*^15^N values and TLs was significant in all three species of *Rossia*, with a higher steep increase in *R. moelleri*, followed by *R. palpebrosa* and *R. megaptera*. Generally ontogenetic increase in *Rossia* was lower than in squids^42,53,76,77,83,84^, but similar, or more pronounced, than in octopods^32,53,78^.

Ontogenetic isotopic niche decrease is common in cephalopods, including *R. palpebrosa*^32,33,41,42,84^ and *R. moelleri*. On the other hand, *R. megaptera* demonstrated ontogenetic niche increase, similar to *Vampyroteuthis infernalis*, a deep-sea cephalopod with unique diet and life style^54^, but this is rarely found in ‘typical’ predatory cephalopods^32,83^. Within the Arctic, isotopic niches of all *Rossia* were narrower than of squid *Gonatus fabricii* (which was the widest among Arctic invertebrates^42^) and of shrimp *Pandalus borealis* and fishes due to their higher degree of opportunism in diet^60,64–67^.

How the life style is a means to reducing competition? *Rossia* beaks had high range of differences in *δ*^13^C values (4.1–4.6‰; Table 2), as was previously found in polar squids^42,53,77,85^, compared to warm-water ones^58,76,81–84,86^. Contrary to majority of the studied squids and octopods with ontogenetic increase of *δ*^13^C values^32,33,42,53,76,84^, *δ*^13^C values remain the same throughout the ontogenesis in *R. palpebrosa*, suggesting it does not migrate during ontogenesis. On the other hand, *δ*^13^C values decreased in *R. megaptera* and *R. moelleri* suggesting they migrate during ontogenesis, despite a nekto-benthic life style. Significantly higher TLs in the Barents Sea than in East Greenland (*R. megaptera*) and in the Kara Sea (*R. moelleri*) further suggest these species migrate during ontogenesis: their diets were less varying between regions, than in *R. palpebrosa*. Differences in TLs among regions were not found in other studied Arctic cephalopods^41,42^.

As nekto-benthic species, Arctic sepiolids were supposed to have higher *δ*^13^C values than pelagic Arctic squid. However, *Rossia moelleri*, the shallowest living species, had *δ*^13^C values similar to the Arctic squid *G. fabricii*^42^, and significantly lower than *R. papebrosa* and *R. megaptera*, suggesting a different, relatively more pelagic life style.

Differences in the widths of isotopic niches between sexes were found in *R. megaptera* and *R. moelleri*: females had large niches, and niches of males were almost completely within the isotopic niche of females. However, and in accordance with Golikov et al.^41^, no differences were found in isotopic niche widths of *R. palpebrosa* between sexes. *Rossia megaptera* and *R. moelleri*, and squid species which demonstrated the same pattern of niche differences^83,86^ all had more pronounced sexual differences in body sizes, than *R. palpebrosa*. However, niche overlap between sexes was decreasing during ontogenesis in squids^83,86^, unlike in the studied species of the genus *Rossia*.

Our data suggest that predicted climate changes in the Arctic would: (1) not significantly change the situation for *R. moelleri*, even if its range decreases due to its Arctic affiliation; (2) create more favourable conditions for niche width increase in *R. megaptera* in the Barents Sea, where it is currently in disadvantage, inhabiting only the warmer, western part, and strengthen its advantage in West Greenland; (3) not significantly decrease abundance of *R. palpebros*a due to its plasticity, as this is the most widespread *Rossia* in the Arctic, which has the most varying diet and the widest habitable diapason of temperatures.

## Conclusion

Three sympatric species of cephalopods of the genus *Rossia* (widespread boreal Arctic *R. palpebrosa*, high boreal *R. megaptera* and Arctic endemic *R. moelleri*) with seemingly similar sizes and hunting behaviour, which live together to a degree they can be sampled all together in one trawl catch, were found to have seven traits related to resource and habitat utilization: no trait was shared by all three species, and high boreal *R. megaptera* and Arctic endemic *R. moelleri* shared three traits with each other, while both *R. megaptera* and *R. moelleri* shared only two unique traits each with widespread boreal-Arctic *R. palpebrosa*. No crossing pattern was formed from traits with each species having unique strategy of resource and habitat utilization. Such a fine level of competition-avoidance is not easily detected, these traits were only highlighted by SIA and its applications when applied to the sample including all ontogenetic stages and both sexes in largely equal ratio and missed by ‘classical’ methods, such as e.g. stomach contents or distributional analyses. Further SIA studies of sympatric species based on all-ontogenetic samples with equal sex ratio are recommended to increase our understanding of inter- and intraspecific competition, and thus complex trophic webs under natural conditions.

## Supplementary Information

Supplementary Information.

## Data Availability

All relevant data are included in the paper and/or in the supplementary information.

## References

[1] Gause GF (1934). The Struggle for Existence.

[2] Hutchinson GE (1957). Concluding remarks. Cold Spring Harb. Symp. Quant. Biol..

[3] Volterra V (1928). Variations and fluctuations of the number of individuals in marine intertidal species living together. J. Conseil..

[4] Darwin C (1859). On the Origin of Species by Natural Selection, or the Preservation of Favoured Races in the Struggle for Life.

[5] Hardin G (1960). The competitive exclusion principle. Science.

[6] Alley TR (1982). Competition theory, evolution, and the concept of an ecological niche. Acta Biotheor..

[7] Chase JM, Leibold MA (2003). Ecological Niches: Linking Classical and Contemporary Approaches.

[8] Pianka ER, May RM (1976). Competition and niche theory. Theoretical Ecology: Principles and Applications.

[9] Gerking SD (1994). The Feeding Ecology of Fish.

[10] Ross ST (1986). Resource partitioning in fish assemblages: a review of field studies. Copeia.

[11] Persson L (1985). Asymmetrical competition: are larger animals competitively superior?. Am. Nat..

[12] Boecklen WJ, Yarnes CT, Cook BA, James AC (2011). On the use of stable isotopes in trophic ecology. Annu. Rev. Ecol. Evol. Syst..

[13] Layman CA (2012). Applying stable isotopes to examine food-web structure: an overview of analytical tools. Biol. Rev. Camb. Philos..

[14] Bearhop S, Adams CE, Waldron S, Fuller RA, MacLeod H (2004). Determining trophic niche width: a novel approach using stable isotope analysis. J. Anim. Ecol..

[15] Jackson AL, Inger R, Parnell AC, Bearhop S (2011). Comparing isotopic niche widths among and within communities: SIBER-stable isotope Bayesian ellipses in R. J. Anim. Ecol..

[16] Layman CA, Arrington DA, Montaña CG, Post DM (2007). Can stable isotope ratios provide for community-wide measures of trophic structure?. Ecology.

[17] Newsome SD, del Rio CM, Bearhop S, Phillips DL (2007). A niche for isotopic ecology. Front. Ecol. Environ..

[18] Hette-Tronquart N (2019). Isotopic niche is not equal to trophic niche. Ecol. Lett..

[19] Parnell CA (2013). Bayesian stable isotope mixing models. Environmetrics.

[20] Phillips DL (2014). Best practices for use of stable isotope mixing models in food-web studies. Can. J. Zool..

[21] Knickle DC, Rose GA (2014). Dietary niche partitioning in sympatric gadid species in coastal Newfoundland: evidence from stomachs and C-N isotopes. Environ. Biol. Fish..

[22] Simpson SJ, Sims DW, Trueman CM (2019). Ontogenetic trends in resource partitioning and trophic geography of sympatric skates (Rajidae) inferred from stable isotope composition across eye lenses. Mar. Ecol. Prog. Ser..

[23] Bearhop S, Phillips RA, McGill R, Cherel Y, Dawson DA, Croxall JP (2006). Stable isotopes indicate sex-specific and long-term individual foraging specialisation in diving seabirds. Mar. Ecol. Prog. Ser..

[24] Young HS, McCauley DJ, Dirzo R, Dunbar RB, Shaffer SA (2010). Niche partitioning among and within sympatric tropical seabirds revealed by stable isotope analysis. Mar. Ecol. Prog. Ser..

[25] Botta S (2018). Isotopic niche overlap and partition among three Antarctic seals from the Western Antarctic Peninsula. Deep-Sea Res..

[26] Kiszka J, Simon-Bouhet B, Martinez L, Pusineri C, Richard P, Ridoux V (2011). Ecological niche segregation within a community of sympatric dolphins around a tropical island. Mar. Ecol. Prog. Ser..

[27] Ogloff WR, Yurkowski DJ, Davoren GK, Ferguson SH (2019). Diet and isotopic niche overlap elucidate competition potential between seasonally sympatric phocids in the Canadian Arctic. Mar. Biol..

[28] Dubois S, Orvain F, Marin-Léal JC, Ropert M, Lefebvre S (2007). Small-scale spatial variability of food partitioning between cultivated oysters and associated suspension feeding species, as revealed by stable isotopes. Mar. Ecol. Prog. Ser..

[29] Karlson AML, Gorokhova E, Elmgren R (2015). Do deposit-feeders compete? Isotopic niche analysis of an invasion in a species-poor system. Sci. Rep..

[30] Taupp T, Hellmann C, Gergs R, Winkelmann C, Wetzel MA (2017). Life under exceptional conditions—isotopic niches of benthic invertebrates in the estuarine maximum turbidity zone. Estuar. Coast..

[31] Bennice CO, Rayburn AR, Brooks WR, Hanlon RT (2019). Fine-scale habitat partitioning facilitates sympatry between two octopus species in a shallow Florida lagoon. Mar. Ecol. Prog. Ser..

[32] Matias RS (2019). Show your beaks and we tell you what you eat: different ecology in sympatric Antarctic benthic octopods under a climate change context. Mar. Environ. Res..

[33] Rosas-Luis R, Navarro J, Sánchez P, del Río JL (2016). Assessing the trophic ecology of three sympatric squid in the marine ecosystem off the Patagonian Shelf by combining stomach content and stable isotopic analyses. Mar. Biol. Res..

[34] Boyle PR, Rodhouse PG (2005). Cephalopods: Ecology and Fisheries.

[35] Rodhouse PG, Nigmatullin ChM (1996). Role as consumers. Philos. Trans. R. Soc. B.

[36] Jereb, P. & Roper, C.F.E. *Cephalopods of the world. An annotated and illustrated catalogue of cephalopod species known to date. Volume 1. Chambered nautiluses and sepioids (Nautilidae, Sepiidae, Sepiolidae, Sepiadariidae, Idiosepiidae and Spirulidae). FAO Species Catalogue for Fishery Purposes, No. 4*. Rome: FAO (2005).

[37] Golikov AV, Sabirov RM, Lubin PA, Jørgensen LL (2013). Changes in distribution and range structure of Arctic cephalopods due to climatic changes of the last decades. Biodiversity.

[38] Nesis KN, Kafanov AI (1987). Cephalopod mollusks of the Arctic Ocean and its seas. Fauna and Distribution of Molluscs: North Pacific and Arctic Basin.

[39] Xavier JC (2018). A review on the biodiversity, distribution and trophic role of cephalopods in the Arctic and Antarctic marine ecosystems under a changing ocean. Mar. Biol..

[40] Golikov AV (2019). Reproductive biology and ecology of the boreoatlantic armhook squid *Gonatus fabricii* (Cephalopoda: Gonatidae). J. Mollus. Stud..

[41] Golikov AV (2019). Food spectrum and trophic position of an Arctic cephalopod, *Rossia palpebrosa* (Sepiolida), inferred by stomach contents and stable isotope (*δ*^13^C and *δ*^15^N) analyses. Mar. Ecol. Prog. Ser..

[42] Golikov AV (2018). Ontogenetic changes in stable isotope (*δ*^13^C and *δ*^15^N) values in squid *Gonatus fabricii* (Cephalopoda) reveal its important ecological role in the Arctic. Mar. Ecol. Prog. Ser..

[43] Golikov AV, Sabirov RM, Lubin PA (2017). First assessment of biomass and abundance of cephalopods *Rossia palpebrosa* and *Gonatus fabricii* in the Barents Sea. J. Mar. Biol. Assoc. UK.

[44] Nesis KN (1985). Oceanic Cephalopods: Distribution, Life Forms, Evolution.

[45] Overland JE, Wang M, Walsh JE, Stroeve JC (2014). Future Arctic climate changes: adaptation and mitigation time scales. Earth’s Future.

[46] Dalpadado P (2020). Climate effects on temporal and spatial dynamics of phytoplankton and zooplankton in the Barents Sea. Prog. Oceanogr..

[47] Laidre KL (2015). Arctic marine mammal population status, sea ice habitat loss, and conservation recommendations for the 21st century. Conserv. Biol..

[48] Mercer, M.C. *Systematics of the Sepiolid Squid Rossia Owen 1835 in Canadian Waters with a Preliminary Review of the Genus and Notes on Biology* (MSc thesis). St. Johns: Memorial University of Newfoundland (1968).

[49] Golikov, A.V. *Distribution and reproductive biology of ten-armed cephalopods (Sepiolida, Teuthida) in the Barents Sea and adjacent areas* (PhD thesis). Moscow: Moscow State University (2015) (**in Russian**).

[50] Golikov, A.V., Sabirov, R.M., Gudmundsson, G. *Cephalopoda (Smokkdýr), Rossia megaptera Verrill, 1881*. (2018). http://www.ni.is/biota/animalia/mollusca/cephalopoda/rossia-megaptera. Accessed 04 June 2020.

[51] Golikov AV, Morov AR, Sabirov RM, Lubin PA, Jørgensen LL (2013). Functional morphology of reproductive system of *Rossia palpebrosa* (Cephalopoda, Sepiolida) in Barents Sea. Proc. Kazan Univ. Nat. Sci. Ser..

[52] Cherel Y, Ducatez S, Fontaine C, Richard P, Guinet C (2008). Stable isotopes reveal the trophic position and mesopelagic fish diet of female southern elephant seals breeding on the Kerguelen Islands. Mar. Ecol. Prog. Ser..

[53] Cherel Y, Hobson KA (2005). Stable isotopes, beaks and predators: a new tool to study the trophic ecology of cephalopods, including giant and colossal squids. Proc. R. Soc. B..

[54] Golikov AV (2019). The first global deep-sea stable isotope assessment reveals the unique trophic ecology of Vampire Squid *Vampyroteuthis infernalis* (Cephalopoda). Sci. Rep..

[55] Cherel Y, Fontaine C, Jackson GD, Jackson CH, Richard P (2009). Tissue, ontogenic and sex-related differences in *δ*^13^C and *δ*^15^N values of the oceanic squid *Todarodes filippovae* (Cephalopoda: Ommastrephidae). Mar. Biol..

[56] Zar JH (2010). Biostatistical Analysis.

[57] Ruiz-Cooley RI, Garcia KY, Hetherington ED (2011). Effects of lipid removal and preservatives on carbon and nitrogen stable isotope ratios of squid tissues: implications for ecological studies. J. Exp. Mar. Biol. Ecol..

[58] Hobson KA, Cherel Y (2006). Isotopic reconstruction of marine food webs using cephalopod beaks: new insight from captively raised *Sepia officinalis*. Can. J. Zool..

[59] Post DM (2002). Using stable isotopes to estimate trophic position: models, methods and assumptions. Ecology.

[60] Hobson KA, Fisk A, Karnovsky N, Holst M, Gagnon JM, Fortier M (2002). A stable isotope (*δ*^13^C, *δ*^15^N) model for the North Water food web: implications for evaluating trophodynamics and the flow of energy and contaminants. Deep-Sea Res. II.

[61] Van der Zanden MJ, Cabana G, Rasmussen JB (1997). Comparing trophic position of freshwater fish calculated using stable nitrogen isotope ratios (*δ*^15^N) and literature dietary data. Can. J. Fish. Aquat. Sci..

[62] Hussey NE (2014). Rescaling the trophic structure of marine food webs. Ecol. Lett..

[63] Hussey NE (2014). Corrigendum to Hussey et al. (2014). Ecol. Lett..

[64] Linnebjerg JF (2016). Deciphering the structure of the West Greenland marine food web using stable isotopes (*δ*^13^C, *δ*^15^N). Mar. Biol..

[65] Søreide JE, Carroll ML, Hop H, Ambrose WG, Hegseth EN, Falk-Petersen S (2013). Sympagic-pelagic-benthic coupling in Arctic and Atlantic waters around Svalbard revealed by stable isotopic and fatty acid tracers. Mar. Biol. Res..

[66] Sokolowski A, Szczepanska A, Richard P, Kedra M, Wolowicz M, Weslawski JM (2014). Trophic structure of the macrobenthic community of Hornsund, Spitsbergen, based on the determination of stable carbon and nitrogen isotopic signatures. Polar Biol..

[67] Tamelander T, Renaud PE, Hop H, Carroll ML, Ambrose WG, Hobson KA (2006). Trophic relationships and pelagic-benthic coupling during summer in the Barents Sea marginal ice zone, revealed by stable carbon and nitrogen isotope measurements. Mar. Ecol. Prog. Ser..

[68] R Development Core Team. *R: A Language and Environment for Statistical Computing.* R Foundation for Statistical Computing, Vienna. (2019). http://www.r-project.org/. Accessed 04 June 2020.

[69] Syväranta J, Lensu A, Marjomaki TJ, Oksanen S, Jones RI (2013). An empirical evaluation of the utility of convex hull and standard ellipse areas for assessing population niche widths from stable isotope data. PLoS ONE.

[70] Langton RW (1982). Diet overlap between Atlantic cod, *Gadus morphua*, silver hake, *Merluccius bilinearis*, and fifteen other northwest Atlantic finfish. Fish. B NOAA.

[71] Parnell, C.A. *simmr: A Stable Isotope Mixing Model. Version 0.4.1*. (2019). https://cran.r-project.org/web/packages/simmr/. Accessed 04 June 2020.

[72] Smith JA, Mazumder D, Suthers IM, Taylor MD (2013). To fit or not to fit: evaluating stable isotope mixing models using simulated mixing polygons. Methods Ecol. Evol..

[73] Hammer Ø, Harper DAT, Ryan PD (2001). PAST: paleontological statistics software package for education and data analysis. Palaeontol. Electron..

[74] Gruber N (1999). Spatiotemporal patterns of carbon-13 in the global surface oceans and the oceanic Suess effect. Glob. Biogeochem. Cycl..

[75] Yurkowski DJ, Hussey NE, Ferguson SH, Fisk AT (2018). A temporal shift in trophic diversity among a predator assemblage in a warming Arctic. R. Soc. Open. Sci..

[76] Guerra A, Rodríguez-Navarro AB, González AF, Romanek CS, Álvarez-Lloret P, Pierce GJ (2010). Life-history traits of the giant squid *Architeuthis dux* revealed from stable isotope signatures recorded in beaks. ICES J. Mar. Sci..

[77] Queirós JP, Cherel Y, Ceia FR, Hilário A, Roberts J, Xavier JC (2018). Ontogenic changes in habitat and trophic ecology in the Antarctic squid *Kondakovia longimana* derived from isotopic analysis on beaks. Polar Biol..

[78] Queirós JP, Fenwick M, Stevens DW, Cherel Y, Ramos JA, Xavier JC (2020). Ontogenetic changes in habitat and trophic ecology of the giant Antarctic octopus *Megaleledone setebos* inferred from stable isotope analyses in beaks. Mar. Biol..

[79] Hansen HJ, Hedeholm RB, Sünksen K, Christensen JT, Grønkjær P (2012). Spatial variability of carbon (*δ*^13^C) and nitrogen (*δ*^15^N) stable isotope ratios in an Arctic marine food web. Mar. Ecol. Prog. Ser..

[80] Cherel Y, Ridoux V, Spitz J, Richard P (2009). Stable isotopes document the trophic structure of a deep-sea cephalopod assemblage including giant octopod and giant squid. Biol. Lett..

[81] Chouvelon T (2012). Revisiting the use of *δ*^15^N in meso-scale studies of marine food webs by considering spatio-temporal variations in stable isotopic signatures—the case of an open ecosystem: the Bay of Biscay (North-East Atlantic). Prog. Oceanogr..

[82] Das K, Lepoint G, Leroy Y, Bouquegneau JM (2003). Marine mammals from the southern North Sea: feeding ecology data from *δ*^13^C and *δ*^15^N measurements. Mar. Ecol. Prog. Ser..

[83] Gong Y, Ruiz-Cooley RI, Hunsicker ME, Li Y, Chen X (2018). Sexual dimorphism in feeding apparatus and niche partitioning in juvenile jumbo squid *Dosidicus gigas*. Mar. Ecol. Prog. Ser..

[84] Trasviña-Carrillo LD, Hernández-Herrera A, Torres-Rojas YE, Galván-Magaña F, Sánchez-González A, Aguíñiga-García S (2018). Spatial and trophic preferences of jumbo squid *Dosidicus gigas* (D’Orbigny, 1835) in the central Gulf of California: ecological inferences using stable isotopes. Rapid Commun. Mass. Spectrom..

[85] Guerreiro M (2015). Habitat and trophic ecology of Southern Ocean cephalopods from stable isotope analyses. Mar. Ecol. Prog. Ser..

[86] Kato Y (2016). Stable isotope analysis of the gladius to investigate migration and trophic patterns of the neon flying squid (*Ommastrephes bartramii*). Fish. Res..

