# Supplementary material for: Diet and life history reduce interspecific and intraspecific competition among three sympatric Arctic cephalopods
Source: Sci Rep. 2020 Dec 9;10:21506. doi: 10.1038/s41598-020-78645-z (PMC7726147; doi:10.1038/s41598-020-78645-z)
Supplement: Supplementary file 1 — Supplementary Information. [file 41598_2020_78645_MOESM1_ESM.pdf]

Supplementary materials for the article

Diet and life history reduce interspecific and intraspecific competition among three sympatric Arctic cephalopods

Alexey V. Golikov*, Filipe R. Ceia, Rushan M. Sabirov, Georgii A. Batalin, Martin E. Blicher, Bulat I. Gareev, Gudmundur Gudmundsson, Lis L. Jørgensen, Gazinur Z. Mingazov, Denis V. Zakharov & José C. Xavier

*Corresponding author: golikov.ksu@gmail.com

Supplementary Table 1. Mantle length (ML), sex, ontogenetic stage, stable isotope values ($\delta^{13}C$ and $\delta^{15}N$), estimated trophic level (TL) and collection area of each studied specimen of the genus *Rossia*. BS – Barents Sea, EG – East Greenland, KS – Kara Sea, WG – West Greenland

Species	Area	ML, mm	Sex	Stage	$\delta^{13}C$, ‰	$\delta^{15}N$, ‰	TL
R. palpebrosa	WG	18	Female	Small	–19.2	9.4	3.6
R. palpebrosa	WG	10	Female	Small	–18.9	9.2	3.6
R. palpebrosa	WG	11	Female	Small	–19.1	8.2	3.3
R. palpebrosa	WG	12	Male	Small	–19.4	7.3	3.1
R. palpebrosa	WG	16	Male	Small	–18.5	9.4	3.7
R. palpebrosa	WG	10	Male	Small	–18.9	7.2	3.1
R. palpebrosa	WG	12	Male	Small	–18.6	6.4	2.9
R. palpebrosa	WG	19	Female	Small	–18.6	7.3	3.1
R. palpebrosa	WG	18	Female	Small	–19.3	7.1	3.0
R. palpebrosa	WG	17	Male	Small	–19.2	7.8	3.2
R. palpebrosa	WG	34	Female	Medium	–19.7	9.3	3.6
R. palpebrosa	WG	29	Female	Medium	–17.0	8.1	3.3
R. palpebrosa	WG	24	Female	Medium	–18.3	9.0	3.6
R. palpebrosa	WG	25	Male	Medium	–18.7	8.7	3.5
R. palpebrosa	WG	27	Male	Medium	–17.5	8.6	3.4
R. palpebrosa	WG	38	Female	Medium	–18.5	10.1	3.8
R. palpebrosa	WG	38	Female	Medium	–18.0	6.7	2.9
R. palpebrosa	WG	38	Male	Medium	–18.9	8.5	3.4
R. palpebrosa	WG	35	Male	Medium	–18.8	8.1	3.3
R. palpebrosa	WG	26	Male	Medium	–18.5	6.0	2.7
R. palpebrosa	WG	46	Female	Large	–17.7	9.4	3.7
R. palpebrosa	WG	42	Female	Large	–18.1	9.6	3.7
R. palpebrosa	WG	48	Female	Large	–18.7	11.4	4.2
R. palpebrosa	WG	50	Female	Large	–19.4	8.4	3.4

R. palpebrosa	WG	56	Female	Large	–18.3	9.2	3.6
R. palpebrosa	WG	51	Male	Large	–19.3	10.2	3.9
R. palpebrosa	WG	56	Male	Large	–18.7	8.7	3.5
R. palpebrosa	WG	46	Male	Large	–18.9	11.2	4.1
R. palpebrosa	WG	45	Male	Large	–18.6	10.1	3.8
R. palpebrosa	WG	44	Male	Large	–18.6	9.6	3.7
R. palpebrosa	EG	31	Female	Medium	–19.4	9.6	3.7
R. palpebrosa	BS	16	Female	Small	–21.2	9.4	3.8
R. palpebrosa	BS	13	Female	Small	–19.4	10.2	4.0
R. palpebrosa	BS	14	Female	Small	–17.1	6.6	3.1
R. palpebrosa	BS	12	Male	Small	–20.3	7.9	3.4
R. palpebrosa	BS	14	Male	Small	–20.8	7.4	3.3
R. palpebrosa	BS	19	Male	Small	–21.6	8.0	3.5
R. palpebrosa	BS	27	Female	Medium	–20.6	8.2	3.5
R. palpebrosa	BS	22	Male	Medium	–20.9	8.6	3.6
R. palpebrosa	BS	40	Female	Medium	–19.3	8.2	3.5
R. palpebrosa	BS	30	Male	Medium	–20.3	9.5	3.9
R. palpebrosa	BS	30	Male	Medium	–20.8	8.3	3.5
R. palpebrosa	BS	32	Female	Medium	–18.6	7.7	3.4
R. palpebrosa	BS	45	Female	Large	–19.7	9.3	3.8
R. palpebrosa	BS	43	Female	Large	–21.1	8.7	3.6
R. palpebrosa	BS	41	Female	Large	–18.9	10.5	4.1
R. palpebrosa	BS	45	Female	Large	–18.7	8.3	3.6
R. palpebrosa	BS	48	Male	Large	–19.7	9.0	3.7
R. palpebrosa	BS	42	Male	Large	–20.5	9.6	3.9
R. megaptera	WG	11	Female	Small	–20.1	7.3	3.1
R. megaptera	WG	10	Female	Small	–18.7	8.3	3.4
R. megaptera	WG	10	Female	Small	–18.1	7.8	3.2
R. megaptera	WG	13	Male	Small	–17.3	7.2	3.1
R. megaptera	WG	10	Male	Small	–19.0	6.8	3.0
R. megaptera	WG	12	Male	Small	–18.4	9.0	3.5
R. megaptera	WG	13	Male	Small	–19.5	8.9	3.5
R. megaptera	WG	16	Male	Small	–18.4	7.2	3.1
R. megaptera	WG	17	Male	Small	–19.5	9.2	3.6
R. megaptera	WG	16	Female	Small	–19.2	6.1	2.8
R. megaptera	WG	21	Female	Medium	–20.2	6.5	2.9
R. megaptera	WG	27	Female	Medium	–20.9	7.7	3.2
R. megaptera	WG	24	Male	Medium	–18.7	9.1	3.6
R. megaptera	WG	26	Male	Medium	–18.7	9.1	3.6
R. megaptera	WG	28	Male	Medium	–19.7	8.9	3.5
R. megaptera	WG	22	Male	Medium	–17.4	7.7	3.2
R. megaptera	WG	39	Female	Medium	–19.3	9.5	3.7

R. megaptera	WG	33	Female	Medium	–17.9	8.5	3.4
R. megaptera	WG	31	Female	Medium	–19.0	9.2	3.6
R. megaptera	WG	35	Male	Medium	–19.5	10.1	3.8
R. megaptera	WG	47	Female	Large	–18.2	7.1	3.1
R. megaptera	WG	41	Female	Large	–18.2	9.5	3.7
R. megaptera	WG	42	Female	Large	–19.6	8.3	3.4
R. megaptera	WG	41	Female	Large	–18.4	9.9	3.8
R. megaptera	WG	41	Female	Large	–17.6	9.1	3.6
R. megaptera	EG	13	Female	Small	–16.6	8.6	3.4
R. megaptera	EG	22	Female	Medium	–16.8	9.2	3.6
R. megaptera	EG	26	Male	Medium	–18.7	6.6	2.9
R. megaptera	EG	25	Male	Medium	–17.6	7.8	3.2
R. megaptera	EG	29	Female	Medium	–18.4	7.9	3.3
R. megaptera	EG	34	Female	Medium	–18.4	6.7	3.0
R. megaptera	EG	35	Male	Medium	–19.3	8.3	3.4
R. megaptera	EG	46	Female	Large	–20.5	9.3	3.6
R. megaptera	BS	17	Female	Small	–18.4	8.5	3.6
R. megaptera	BS	14	Female	Small	–18.7	8.8	3.7
R. megaptera	BS	17	Male	Small	–19.2	8.1	3.5
R. megaptera	BS	18	Male	Small	–18.7	7.6	3.4
R. megaptera	BS	26	Female	Medium	–19.5	9.0	3.7
R. megaptera	BS	21	Male	Medium	–20.7	8.9	3.7
R. megaptera	BS	23	Female	Medium	–19.2	8.2	3.5
R. megaptera	BS	32	Female	Medium	–20.6	8.3	3.6
R. megaptera	BS	26	Male	Medium	–18.9	8.9	3.7
R. megaptera	BS	28	Male	Medium	–18.3	8.1	3.5
R. megaptera	BS	42	Female	Large	–21.0	8.3	3.6
R. megaptera	BS	42	Female	Large	–20.6	8.7	3.7
R. moelleri	BS	9	Female	Small	–19.6	6.5	3.1
R. moelleri	BS	31	Female	Medium	–21.0	8.9	3.7
R. moelleri	BS	39	Female	Medium	–20.7	9.0	3.7
R. moelleri	BS	40	Female	Medium	–20.2	9.2	3.8
R. moelleri	BS	38	Female	Medium	–20.9	9.6	3.9
R. moelleri	BS	21	Male	Medium	–20.2	9.5	3.9
R. moelleri	BS	33	Male	Medium	–21.9	7.9	3.4
R. moelleri	BS	39	Male	Medium	–21.9	8.7	3.7
R. moelleri	BS	40	Male	Medium	–20.5	10.4	4.1
R. moelleri	BS	58	Female	Large	–20.6	10.4	4.1
R. moelleri	BS	43	Male	Large	–22.7	8.8	3.7
R. moelleri	BS	46	Male	Large	–21.8	9.9	4.0
R. moelleri	BS	46	Male	Large	–21.9	8.4	3.6
R. moelleri	BS	43	Male	Large	–23.1	9.7	3.9

R. moelleri	BS	67	Female	Very large	–22.4	9.7	3.9
R. moelleri	BS	76	Female	Very large	–22.7	10.6	4.2
R. moelleri	BS	62	Female	Very large	–22.7	10.2	4.0
R. moelleri	KS	12	Male	Small	–22.1	8.1	3.3
R. moelleri	KS	24	Female	Medium	–23.0	7.1	3.1
R. moelleri	KS	26	Female	Medium	–22.6	7.3	3.1
R. moelleri	KS	28	Female	Medium	–22.5	9.2	3.6
R. moelleri	KS	26	Female	Medium	–23.2	8.5	3.4
R. moelleri	KS	40	Female	Medium	–22.9	9.2	3.6
R. moelleri	KS	21	Male	Medium	–22.7	8.8	3.5
R. moelleri	KS	24	Male	Medium	–22.8	9.1	3.6
R. moelleri	KS	28	Male	Medium	–22.0	7.6	3.2
R. moelleri	KS	36	Male	Medium	–23.4	10.6	4.0
R. moelleri	KS	36	Male	Medium	–22.4	9.1	3.6
R. moelleri	KS	40	Male	Medium	–21.1	9.4	3.7
R. moelleri	KS	42	Female	Large	–22.1	8.9	3.5
R. moelleri	KS	58	Female	Large	–22.8	9.6	3.7
R. moelleri	KS	42	Male	Large	–23.7	8.7	3.5
R. moelleri	KS	46	Male	Large	–22.5	9.8	3.8
R. moelleri	KS	43	Male	Large	–22.0	10.3	3.9
R. moelleri	KS	43	Male	Large	–22.8	10.3	3.9
R. moelleri	KS	42	Male	Large	–22.7	9.1	3.6
R. moelleri	KS	68	Female	Very large	–21.5	10.1	3.9
R. moelleri	KS	69	Female	Very large	–22.0	11.3	4.2
R. moelleri	KS	67	Female	Very large	–22.8	10.8	4.0

Supplementary Table 2. Mantle length (ML), values of δ^{13}C and δ^{15}N and estimated trophic level (TL) in the studied species of the genus *Rossia* from the Barents Sea. Values are minimum – maximum (mean ± SE)

Stage/ parameter	All	Small	Medium	Large	Very large
			Rossia palpebrosa		
n	18	6	6	6	–
ML, mm	12–48 (29.6 ± 3.0)	12–19 (14.7 ± 1.0)	22–40 (30.2 ± 2.4)	41–48 (44.0 ± 1.0)	–
δ^{13}C, ‰	–21.6– –17.1 (–20.0 ± 0.3)	–21.6– –17.1 (–20.0 ± 0.7)	–20.9– –18.6 (–20.1 ± 0.4)	–21.1– –18.7 (–19.8 ± 0.4)	–
δ^{15}N, ‰	6.6–10.5 (8.6 ± 0.2)	6.6–10.2 (8.3 ± 0.5)	7.7–9.5 (8.4 ± 0.3)	8.3–10.5 (9.2 ± 0.3)	–
TL	3.1–4.1 (3.6 ± 0.1)	3.1–4.0 (3.5 ± 0.1)	3.4–3.9 (3.6 ± 0.1)	3.6–4.1 (3.8 ± 0.1)	–
Stage/ parameter	All	Small	Medium	Large	Very large
			Rossia megaptera		
n	12	4	6	2	–
ML, mm	14–42 (25.5 ± 2.7)	14–18 (16.5 ± 0.9)	21–32 (26.0 ± 1.6)	42	–
δ^{13}C, ‰	–21.0– –18.3 (–19.5 ± 0.3)	–19.2– –18.4 (–18.7 ± 0.2)	–20.7– –18.3 (–19.5 ± 0.4)	–21.0– –20.6 (–20.8 ± 0.2)	–
δ^{15}N, ‰	7.7–8.9 (8.5 ± 0.1)	7.6–8.8 (8.3 ± 0.3)	8.1–9.0 (8.6 ± 0.2)	8.3–8.7 (8.5 ± 0.2)	–
TL	3.4–3.7 (3.6 ± 0.03)	3.4–3.7 (3.5 ± 0.1)	3.5–3.7 (3.6 ± 0.04)	3.6–3.7 (3.6 ± 0.05)	–
Stage/ parameter	All	Small	Medium	Large	Very large
			Rossia moelleri		
n	17	1	8	5	3
ML, mm	9–76 (43.0 ± 3.9)	9	21–40 (35.1 ± 2.3)	43–58 (47.2 ± 2.8)	62–76 (68.3 ± 4.1)
δ^{13}C, ‰	–23.1– –19.6 (–21.5 ± 0.3)	–19.6	–22.0– –20.2 (–20.9 ± 0.2)	–23.1– –20.6 (–22.0 ± 0.4)	–22.7– –22.4 (–22.6 ± 0.1)
δ^{15}N, ‰	6.5–10.6 (9.3 ± 0.3)	6.5	7.9–10.4 (9.2 ± 0.3)	8.4–10.4 (9.5 ± 0.4)	9.7–10.6 (10.2 ± 0.3)
TL	3.1–4.2 (3.8 ± 0.1)	3.1	3.4–4.1 (3.8 ± 0.1)	3.6–4.1 (3.9 ± 0.1)	3.9–4.2 (4.0 ± 0.1)

Supplementary Table 3. Mantle length (ML), values of $\delta^{13}C$ and $\delta^{15}N$ and estimated trophic level (TL) in the studied species of the genus *Rossia* from West Greenland. Values are minimum – maximum (mean ± SE)

Stage/ parameter	*Rossia palpebrosa*			
	All	Small	Medium	Large
n	30	10	10	10
ML, mm	10–56 (31.4 ± 2.7)	10–19 (14.3 ± 1.2)	24–38 (31.4 ± 1.8)	42–56 (48.4 ± 1.5)
$\delta^{13}C$, ‰	–19.7– –17.0 (–18.7 ± 0.1)	–19.4– –18.5 (–19.0 ± 0.1)	–19.7– –17.0 (–18.4 ± 0.2)	–19.4– –17.7 (–18.6 ± 0.2)
$\delta^{15}N$, ‰	6.0–11.4 (8.7 ± 0.2)	6.4–9.4 (7.9 ± 0.3)	6.0–10.1 (8.3 ± 0.4)	8.5–11.4 (9.8 ± 0.3)
TL	2.7–4.2 (3.5 ± 0.1)	2.9–3.7 (3.3 ± 0.1)	2.7–3.8 (3.4 ± 0.1)	3.4–4.2 (3.8 ± 0.1)
Stage/ parameter	*Rossia megaptera*			
	All	Small	Medium	Large
n	25	10	10	5
ML, mm	10–47 (25.0 ± 2.4)	10–17 (12.8 ± 0.9)	21–39 (28.6 ± 1.9)	41–47 (42.4 ± 1.2)
$\delta^{13}C$, ‰	–20.9– –17.3 (–18.7 ± 0.2)	–20.1– –17.3 (–18.8 ± 0.3)	–20.9– –17.4 (–19.1 ± 0.3)	–19.6– –17.6 (–18.4 ± 0.3)
$\delta^{15}N$, ‰	6.1–10.1 (8.3 ± 0.2)	6.1–9.2 (7.8 ± 0.3)	6.5–10.1 (8.6 ± 0.3)	7.1–9.9 (8.8 ± 0.5)
TL	2.8–3.8 (3.4 ± 0.1)	2.8–3.6 (3.2 ± 0.1)	2.9–3.8 (3.4 ± 0.1)	3.1–3.8 (3.5 ± 0.1)

Supplementary Table 4. Mantle length (ML), values of $\delta^{13}C$ and $\delta^{15}N$ and estimated trophic level (TL) in the studied species of the genus *Rossia* from East Greenland (*R. palpebrosa* and *R. megaptera*) and the Kara Sea (*R. moelleri*). Values are minimum – maximum (mean ± SE)

Stage/ parameter	*Rossia palpebrosa*				
	All	Small	Medium	Large	Very large
n	1	–	1	–	–
ML, mm	31	–	31	–	–
$\delta^{13}C$, ‰	–19.4	–	–19.4	–	–
$\delta^{15}N$, ‰	9.7	–	9.7	–	–
TL	3.7	–	3.7	–	–
**Stage/ parameter**	***Rossia megaptera***				
	All	Small	Medium	Large	Very large
n	8	1	6	1	–
ML, mm	13–46 (28.8 ± 3.5)	13	22–35 (28.5 ± 2.1)	46	–
$\delta^{13}C$, ‰	–20.5– –16.6 (–18.3 ± 0.5)	–16.6	–19.3– –16.8 (–18.2 ± 0.4)	–20.5	–
$\delta^{15}N$, ‰	6.6–9.3 (8.1 ± 0.4)	8.6	6.6–9.2 (7.7 ± 0.4)	9.3	–
TL	2.9–3.6 (3.3 ± 0.1)	3.4	2.9–3.8 (3.4 ± 0.1)	3.6	–
**Stage/ parameter**	***Rossia moelleri***				
	All	Small	Medium	Large	Very large
n	22	1	11	7	3
ML, mm	12–69 (39.1 ± 3.3)	12	21–40 (29.9 ± 2.1)	42–58 (45.1 ± 2.2)	67–69 (68.0 ± 0.6)
$\delta^{13}C$, ‰	–23.7– –21.1 (–22.5 ± 0.1)	–22.1	–23.4– –21.1 (–22.6 ± 0.2)	–23.7– –22.0 (–22.7 ± 0.2)	–22.8– –21.5 (–22.1 ± 0.4)
$\delta^{15}N$, ‰	7.1–11.3 (9.2 ± 0.2)	8.1	7.1–10.6 (8.7 ± 0.3)	8.8–10.4 (9.6 ± 0.3)	10.1–11.3 (10.7 ± 0.3)
TL	3.1–4.2 (3.6 ± 0.1)	3.3	3.1–4.0 (3.5 ± 0.1)	3.5–3.9 (3.7 ± 0.1)	3.9–4.2 (4.0 ± 0.1)

Supplementary Table 5. Ontogenetic patterns (via linear regressions) and comparison of δ^{13}C and δ^{15}N values and estimated trophic level (TL) within the studied species of the genus *Rossia*. Kruskal-Wallis *H* and Mann-Whitney *U* tests for between-groups comparisons are provided in the table. Significant *p*-values are in **bold**. Sample size per species, area and stage were already presented in Table 2, Supplementary Tables 1–4. BS – Barents Sea, EG – East Greenland, KS – Kara Sea, WG – West Greenland

Areas*		*Rossia palpebrosa*	
	δ^{13}C, ‰	δ^{15}N, ‰	TL
All	δ^{13}C = 0.0108ML – 19.484 (n = 49, r^2 = 0.02, p = 0.32)<br>$H_{2,49}$ = 1.56, p = 0.45	δ^{15}N = 0.0421ML + 7.3821 (n = 49, r^2 = 0.24, p = **0.0004**)<br>$H_{2,49}$ = 14.84, p = **0.0006**<br>Small vs. Medium: U = 99, p = 0.57<br>Small vs. Large: U = 39.5, p = **0.0027**<br>Medium vs. Large: U = 50, p = **0.0062**	TL = 0.0107ML + 3.2036 (n = 49, r^2 = 0.21, p = **0.0011**)<br>$H_{2,49}$ = 15.20, p = **0.0005**<br>Small vs. Medium: U = 106, p = 0.86<br>Small vs. Large: U = 39, p = **0.0024**<br>Medium vs. Large: U = 47, p = **0.0039**
BS	δ^{13}C = 0.0125ML – 20.8333 (n = 18, r^2 = 0.02, p = 0.58)<br>$H_{2,18}$ = 0.75, p = 0.69	δ^{15}N = 0.0269ML + 7.8333 (n = 18, r^2 = 0.12, p = 0.15)<br>$H_{2,18}$ = 4.35, p = 0.11	TL = 0.0071ML + 3.4298 (n = 18, r^2 = 0.12, p = 0.15)<br>$H_{2,18}$ = 4.40, p = 0.11
WG	δ^{13}C = 0.0062ML – 18.855 (n = 30, r^2 = 0.02, p = 0.41)<br>$H_{2,30}$ = 5.11, p = 0.08	δ^{15}N = 0.0488ML + 7.138 (n = 30, r^2 = 0.30, p = **0.0017**)<br>$H_{2,30}$ = 11.26, p = **0.0036**<br>Small vs. Medium: U = 41, p = 0.99<br>Small vs. Large: U = 9.5, p = **0.0075**<br>Medium vs. Large: U = 15.5, p = **0.0304**	TL = 0.0128ML + 3.0574 (n = 30, r^2 = 0.30, p = **0.0017**)<br>$H_{2,30}$ = 11.87, p = **0.0026**<br>Small vs. Medium: U = 39.5, p = 0.99<br>Small vs. Large: U = 9.5, p = **0.0070**<br>Medium vs. Large: U = 14, p = **0.0209**
Areas		*Rossia megaptera*	
	δ^{13}C, ‰	δ^{15}N, ‰	TL
All	δ^{13}C = –0.0219ML – 18.354 (n = 45, r^2 = 0.05, p = 0.13)<br>$H_{2,45}$ = 1.11, p = 0.57	δ^{15}N = 0.0282ML + 7.5713 (n = 45, r^2 = 0.11, p = **0.0287**)<br>$H_{2,45}$ = 4.20, p = 0.12	TL = 0.0073ML + 3.2255 (n = 45, r^2 = 0.09, p = **0.0497**)<br>$H_{2,45}$ = 3.62, p = 0.16
BS	δ^{13}C = –0.0716ML – 17.644 (n = 12, r^2 = 0.47, p = **0.0146**)	δ^{15}N = 0.0035ML + 8.3658 (n = 12, r^2 = 0.01, p = 0.81)	TL = 0.0009ML + 3.57 (n = 12, r^2 = 0.01, p = 0.81)

	$H_{2,12}$ = 4.90, p = 0.09	$H_{2,12}$ = 1.57, p = 0.46	$H_{2,12}$ = 1.57, p = 0.46
EG	δ^{13}C = –0.1209ML – 14.81 (n = 8, r^2 = 0.87, p = **0.0008**)<br>Not enough samples for Kruskal-Wallis H test (n = 1 in small and large specimens)	δ^{15}N = 0.0071ML + 7.8422 (n = 8, r^2 = 0.01, p = 0.87)<br>Not enough samples for Kruskal-Wallis H test (n = 1 in small and large specimens)	TL = 0.0019ML + 3.2427 (n = 8, r^2 = 0.01, p = 0.87)<br>Not enough samples for Kruskal-Wallis H test (n = 1 in small and large specimens)
WG	δ^{13}C = 0.0055ML – 18.998 (n = 25, r^2 = 0.01, p = 0.73)<br>$H_{2,25}$ = 2.24, p = 0.33	δ^{15}N = 0.0415ML + 7.2669 (n = 25, r^2 = 0.21, p = **0.0223**)<br>$H_{2,25}$ = 4.01, p = 0.14	TL = 0.0109ML + 3.0913 (n = 25, r^2 = 0.21, p = **0.0223**)<br>$H_{2,25}$ = 4.01, p = 0.14
Areas		*Rossia moelleri*	
	δ^{13}C, ‰	δ^{15}N, ‰	TL
All†	δ^{13}C = –0.0107ML – 21.622 (n = 39, r^2 = 0.03, p = 0.15)<br>$H_{2,39}$ = 1.42, p = 0.49	δ^{15}N = 0.0491ML + 7.2438 (n = 39, r^2 = 0.54, p **< 0.0001**)<br>$H_{2,39}$ = 11.90, p = **0.0026**<br>Medium vs. Large: U = 74, p = 0.11<br>Medium vs. Very large: U = 6, p = **0.0013**<br>Large vs. Very large: U = 12, p = **0.0277**	TL = 0.0136ML + 3.1528 (n = 39, r^2 = 0.54, p **< 0.0001**)<br>$H_{2,39}$ = 11.41, p = **0.0033**<br>Medium vs. Large: U = 78, p = 0.15<br>Medium vs. Very large: U = 7, p = **0.0016**<br>Large vs. Very large: U = 11, p = **0.0218**
BS†	δ^{13}C = –0.0423ML – 19.645 (n = 17, r^2 = 0.41, p = **0.0055**)<br>$H_{2,17}$ = 6.95, p = **0.0310**<br>Medium vs. Large: U = 9, p = 0.12<br>Medium vs. Very large: U = 1, p = **0.0189**<br>Large vs. Very large: U = 4, p = 0.37	δ^{15}N = 0.0459ML + 7.2902 (n = 17, r^2 = 0.52, p = **0.0010**)<br>$H_{2,17}$ = 3.94, p = 0.14	TL = 0.0121ML + 3.2869 (n = 17, r^2 = 0.52, p = **0.0010**)<br>$H_{2,17}$ = 3.94, p = 0.14
KS†	δ^{13}C = 0.0075ML – 22.813 (n = 22, r^2 = 0.04, p = 0.39)<br>$H_{2,22}$ = 1.71, p = 0.43	δ^{15}N = 0.0529ML + 7.1655 (n = 22, r^2 = 0.56, p = **0.0001**)<br>$H_{2,22}$ = 7.64, p = **0.0219**<br>Medium vs. Large: U = 22, p = 0.15<br>Medium vs. Very large: U = 1, p = **0.0120**<br>Large vs. Very large: U = 2, p = 0.07	TL = 0.0139ML + 3.0856 (n = 22, r^2 = 0.56, p = **0.0001**)<br>$H_{2,22}$ = 7.64, p = **0.0219**<br>Medium vs. Large: U = 22, p = 0.15<br>Medium vs. Very large: U = 1, p = **0.0120**<br>Large vs. Very large: U = 2, p = 0.07

**R. palpebrosa* from EG is 1 specimen only;

†Small *R. moelleri* are n = 2 only.

Supplementary Table 6. Sexual and geographic comparison of δ^{13}C and δ^{15}N values and estimated trophic level (TL) within the studied species of the genus *Rossia*. Kruskal-Wallis *H* and Mann-Whitney *U* tests for between-groups comparisons are provided in the table. Significant *p*-values are in **bold**. Sample size per species, area, sex and stage were already presented in Table 2, Supplementary Tables 1–4. BS – Barents Sea, EG – East Greenland, KS – Kara Sea, WG – West Greenland

Comparison	Groups	*Rossia palpebrosa* δ^{13}C, ‰	δ^{15}N, ‰	TL
Females vs. Males	All	$U = 238$, $p = 0.22$	$U = 260.5$, $p = 0.45$	$U = 261.5$, $p = 0.46$
	Small	$U = 26$, $p = 0.57$	$U = 23$, $p = 0.38$	$U = 24.5$, $p = 0.46$
	Medium	$U = 26$, $p = 0.38$	$U = 35$, $p = 0.96$	$U = 29.5$, $p = 0.55$
	Large	$U = 26$, $p = 0.61$	$U = 23$, $p = 0.40$	$U = 24$, $p = 0.45$
Areas (BS and WG, as EG has *n* = 1)	All	$U = 81$, $p =$ **0.0002**	$U = 262$, $p = 0.87$	$U = 198$, $p = 0.12$
	Small	$U = 11$, $p =$ **0.0420**	$U = 23$, $p = 0.49$	$U = 16$, $p = 0.14$
	Medium	$U = 5$, $p =$ **0.0047**	$U = 29$, $p = 0.96$	$U = 17.5$, $p = 0.18$
	Large	$U = 6$, $p =$ **0.0075**	$U = 18$, $p = 0.22$	$U = 28$, $p = 0.85$

Comparison	Groups	*Rossia megaptera* δ^{13}C, ‰	δ^{15}N, ‰	TL
Females vs. Males*	All	$U = 229$, $p = 0.69$	$U = 232$, $p = 0.74$	$U = 228$, $p = 0.67$
	Small	$U = 24$, $p = 0.69$	$U = 27$, $p = 0.95$	$U = 27$, $p = 0.95$
	Medium	$U = 52$, $p = 0.61$	$U = 57$, $p = 0.85$	$U = 55$, $p = 0.75$
Areas (BS, EG and WG for all and medium, BS and WG for small and large, as those have *n* = 1)	All	$H_{2,45} = 4.92$, $p = 0.09$	$H_{2,45} = 0.52$, $p = 0.77$	$H_{2,45} = 7.33$, $p =$ **0.0256**<br>BS vs. WG: $U = 82$, $p = 0.09$<br>BS vs. EG: $U = 14$, $p =$ **0.0293**<br>WG vs. EG: $U = 87$, $p = 0.99$
	Small	$U = 17$, $p = 0.73$	$U = 15$, $p = 0.55$	$U = 6$, $p = 0.06$
	Medium	$H_{2,22} = 4.62$, $p = 0.10$	$H_{2,22} = 2.89$, $p = 0.24$	$H_{2,22} = 5.56$, $p = 0.06$
	Large	$U = 1$, $p = 0.10$	$U = 4$, $p = 0.86$	$U = 5$, $p = 0.85$

Comparison	Groups	*Rossia moelleri* δ^{13}C, ‰	δ^{15}N, ‰	TL
Females vs. Males†	All	$U = 176$, $p = 0.71$	$U = 169$, $p = 0.57$	$U = 165$, $p = 0.50$
	Medium	$U = 43$, $p = 0.91$	$U = 36$, $p = 0.49$	$U = 40$, $p = 0.72$
	Large	$U = 10$, $p = 0.60$	$U = 11$, $p = 0.73$	$U = 13$, $p = 0.99$
Areas (BS and KS)	All	$U = 71$, $p =$ **0.0005**	$U = 178$, $p = 0.81$	$U = 115$, $p =$ **0.0429**
	Medium	$U = 2$, $p =$ **0.0003**	$U = 33$, $p = 0.40$	$U = 14$, $p =$ **0.0126**
	Large	$U = 9$, $p = 0.20$	$U = 17$, $p = 0.99$	$U = 9$, $p = 0.20$
	Very large	$U = 3$, $p = 0.70$	$U = 2$, $p = 0.40$	$U = 4$, $p = 0.99$

*All large *R. megaptera* are females;

†All very large *R. moelleri* are females, and small has *n* = 2.

Supplementary Table 7. Overall, geographic and ontogenetic comparison of δ^{13}C and δ^{15}N values and estimated trophic level (TL) among the studied species of the genus *Rossia*. Kruskal-Wallis *H* and Mann-Whitney *U* tests for between-groups comparisons are provided in the table. Significant *p*-values are in **bold**. Sample size per species, area and stage were already presented in Table 2, Supplementary Tables 1–4. BS – Barents Sea, WG – West Greenland

Groups	Parameters		
	δ^{13}C, ‰	δ^{15}N, ‰	TL
3 species, overall	$H_{2,133}$ = 73.74, *p* < **0.0001**	$H_{2,133}$ = 15.77, *p* = **0.0004**	$H_{2,133}$ = 16.78, *p* = **0.0002**
	R. palpebrosa vs. *R. megaptera*: *U* = 975, *p* = 0.34	*R. palpebrosa* vs. *R. megaptera*: *U* = 886, *p* = 0.10	*R. palpebrosa* vs. *R. megaptera*: *U* = 905, *p* = 0.13
	R. palpebrosa vs. *R. moelleri*: *U* = 64, *p* < **0.0001**	*R. palpebrosa* vs. *R. moelleri*: *U* = 673, *p* = **0.0179**	*R. palpebrosa* vs. *R. moelleri*: *U* = 644, *p* = **0.0086**
	R. megaptera vs. *R. moelleri*: *U* = 38, *p* < **0.0001**	*R. megaptera* vs. *R. moelleri*: *U* = 432, *p* < **0.0001**	*R. megaptera* vs. *R. moelleri*: *U* = 420.5, *p* < **0.0001**
2 species, overall, small[*]	*U* = 74, *p* = 0.08	*U* = 116, *p* = 0.89	*U* = 115, *p* = 0.86
3 species, overall, medium	$H_{2,58}$ = 32.20, *p* < **0.0001**	$H_{2,58}$ = 3.79, *p* = 0.15	$H_{2,58}$ = 5.34, *p* = 0.07
	R. palpebrosa vs. *R. megaptera*: *U* = 186, *p* = 0.09		
	R. palpebrosa vs. *R. moelleri*: *U* = 14, *p* < **0.0001**		
	R. megaptera vs. *R. moelleri*: *U* = 14, *p* < **0.0001**		
3 species, overall, large	$H_{2,36}$ = 22.71, *p* < **0.0001**	$H_{2,36}$ = 4.09, *p* = 0.13	$H_{2,36}$ = 5.58, *p* = 0.06
	R. palpebrosa vs. *R. megaptera*: *U* = 64, *p* = 0.98		
	R. palpebrosa vs. *R. moelleri*: *U* = 1, *p* < **0.0001**		
	R. megaptera vs. *R. moelleri*: *U* = 1, *p* = **0.0003**		
3 species, overall, large vs. very large[†]	$H_{2,30}$ = 13.94, *p* = **0.0009**	$H_{2,30}$ = 15.77, *p* = **0.0004**	$H_{2,30}$ = 15.77, *p* = **0.0004**
	R. palpebrosa vs. *R. megaptera*: *U* = 64, *p* = 0.98	*R. palpebrosa* vs. *R. megaptera*: *U* = 886, *p* = 0.10	*R. palpebrosa* vs. *R. megaptera*: *U* = 886, *p* = 0.10
	R. palpebrosa vs. *R. moelleri*: *U* = 1, *p* = **0.0004**	*R. palpebrosa* vs. *R. moelleri*: *U* = 673, *p* = **0.0179**	*R. palpebrosa* vs. *R. moelleri*: *U* = 673, *p* = **0.0179**
	R. megaptera vs. *R. moelleri*: *U* = 1, *p* = **0.0024**	*R. megaptera* vs. *R. moelleri*: *U* = 432, *p* < **0.0001**	*R. megaptera* vs. *R. moelleri*: *U* = 432, *p* < **0.0001**
3 species, BS	$H_{2,47}$ = 16.30, *p* = **0.0003**	$H_{2,47}$ = 10.64, *p* = **0.0049**	$H_{2,47}$ = 12.56, *p* = **0.0018**
	R. palpebrosa vs. *R. megaptera*: *U* = 74, *p* = 0.16	*R. palpebrosa* vs. *R. megaptera*: *U* = 35, *p* = 0.08	*R. palpebrosa* vs. *R. megaptera*: *U* = 28, *p* = 0.09
	R. palpebrosa vs. *R. moelleri*: *U* = 59, *p* = **0.0020**	*R. palpebrosa* vs. *R. moelleri*: *U* = 17, *p* = **0.0245**	*R. palpebrosa* vs. *R. moelleri*: *U* = 16, *p* = 0.06

	R. megaptera vs. *R. moelleri*: $U = 22$, $p =$ **0.0004**	*R. megaptera* vs. *R. moelleri*: $U = 1$, $p =$ **0.0037**	*R. megaptera* vs. *R. moelleri*: $U = 1$, $p =$ **0.0072**
2 species, BS, small*	$U = 4$, $p = 0.11$	$U = 10$, $p = 0.77$	$U = 11$, $p = 0.91$
3 species, BS, medium	$H_{2,20} = 5.39$, $p = 0.07$	$H_{2,20} = 4.82$, $p = 0.09$	$H_{2,20} = 5.03$, $p = 0.08$
2 species, BS, large‡	$U = 1$, $p =$ **0.0097**	$U = 12$, $p = 0.66$	$U = 12$, $p = 0.62$
2 species, BS, large vs. very large†, ‡	$U = 1$, $p =$ **0.0238**	$U = 2$, $p = 0.09$	$U = 2$, $p = 0.09$
2 species, WG§	$U = 330$, $p = 0.45$	$U = 311$, $p = 0.28$	$U = 306$, $p = 0.25$
2 species, WG, small§	$U = 45$, $p = 0.74$	$U = 10$, $p = 0.68$	$U = 44$, $p = 0.67$
2 species, WG, medium§	$U = 27$, $p = 0.09$	$U = 42$, $p = 0.58$	$U = 42$, $p = 0.57$
2 species, WG, large§	$U = 17$, $p = 0.38$	$U = 12$, $p = 0.13$	$U = 11$, $p = 0.10$

***R. moelleri* is not used here, as small *R. moelleri* are $n = 2$ only;

†Very large specimens exist only in *R. moelleri*;

‡Large *R. megaptera* is not used here, as they are $n = 2$ only in BS;

§Only 2 species, *R. palpebrosa* and *R. megaptera*, were used for this study from WG.

Supplementary Table 8. Isotopic niche metrics (TA, SEA_c and SEA_b) for sexes within the studied species of the genus *Rossia*, and respective differences in niche widths (*p* value), and niche overlap. SEA_b values are means ± SD. Significant *p*-values and large overlap values are in **bold**

Species/ parameter	*R. palpebrosa*		*R. megaptera*		*R. moelleri*	
Sexes	Females	Males	Females	Males	Females	Males
n	26	23	26	19	19	20
TA	3.11	3.05	2.85	1.58	3.15	1.98
SEAc	1.02	1.13	1.09	0.62	1.26	0.67
SEAb	1.03 ± 0.21	1.12 ± 0.24	1.08 ± 0.22	0.63 ± 0.15	1.26 ± 0.31	0.67 ± 0.16
Females	–	0.618	–	**0.037**	–	**0.026**
Males	0.382	–	**0.963**	–	**0.974**	–
Overlap, %	**70.8**	**64.0**	54.5	**95.9**	52.1	**98.1**

Supplementary Table 9. Isotopic niche metrics (TA, SEA_c and SEA_b) for ontogenetic groups within the studied species of the genus *Rossia*, and respective differences in niche widths (*p* value), and niche overlap. SEA_b values are means ± SD. Significant *p*-values and large overlap values are in **bold**

Species/parameter	*R. palpebrosa*			*R. megaptera*			*R. moelleri*	
Group	Small	Medium	Large	Small	Medium	Large	Medium	Large and very large
n	16	17	16	15	22	8	19	18
TA	2.82	2.45	1.50	1.94	2.79	1.28	2.10	1.20
SEAc	1.15	0.98	0.68	0.80	0.96	1.07	0.83	0.46
SEAb	0.82 ± 0.22	0.97 ± 0.25	0.72 ± 0.19	0.77 ± 0.21	0.95 ± 0.21	0.73 ± 0.30	0.77 ± 0.19	0.37 ± 0.09
Small	–	0.682	0.3415	–	0.747	0.402	–	–
Medium	0.318	–	0.196	0.253	–	0.2195	–	**0.0075**
Large*	0.6585	0.804	–	0.598	0.7805	–	**0.9925**	–
Overlap, small–medium, %	**63.0** small; **73.9** medium			**76.5** small; **64.1** medium			–	
Overlap, small–large, %	10.1 small; 17.1 large			51.2 small; 38.6 large			–	
Overlap, medium–large*, %	24.1 medium; 34.6 large			**77.7** medium; **69.9** large			12.2 medium; 21.8 large*	

*Large *R. moelleri* (*n* = 12) were pooled with very large (*n* = 6).

Supplementary Table 10. Differences in relative contribution of prey to the diet among the studied species of the genus *Rossia* revealed by Bayesian mixing model SIMMR 0.4.1. χ^2 and Fisher's exact tests are provided in the table, above and below the diagonal, respectively. Significant *p*-values are in **bold**

Species and area*	*R. palpebrosa*, Barents Sea	*R. palpebrosa*, West Greenland	*R. megaptera*, Barents Sea	*R. megaptera*, West Greenland	*R. moelleri*, Barents Sea
R. palpebrosa, Barents Sea	–	χ^2 = 8.74, *d .f.* = 2, *p* = **0.0127**	χ^2 = 3.02, *d .f.* = 2, *p* = 0.22	χ^2 = 7.06, *d .f.* = 2, *p* = **0.0293**	χ^2 = 9.10, *d .f.* = 2, *p* = **0.0106**
R. palpebrosa, West Greenland	*p* = **0.0131**	–	χ^2 = 1.67, *d .f.* = 2, *p* = 0.43	χ^2 = 2.91, *d .f.* = 2, *p* = 0.23	χ^2 = 32.24, *d .f.* = 2, *p* **< 0.0001**
R. megaptera, Barents Sea	*p* = 0.22	*p* = 0.45	–	χ^2 = 3.47, *d .f.* = 2, *p* = 0.18	χ^2 = 20.20, *d .f.* = 2, *p* = < **0.0001**
R. megaptera, West Greenland	*p* = **0.0295**	*p* = 0.25	*p* = 0.18	–	χ^2 = 30.20, *d .f.* = 2, *p* **< 0.0001**
R. moelleri, Barents Sea	*p* = **0.0111**	*p* **< 0.0001**	*p* **< 0.0001**	*p* **< 0.0001**	–

*General comparison among species and areas: χ^2 = 52.30, *d .f.* = 8, *p* **< 0.0001**.

Supplementary Table 11. Differences in relative contribution of prey to the diet on individual level among the studied species of the genus *Rossia*, ontogenetic differences and differences between sexes revealed by Bayesian mixing model SIMMR 0.4.1. χ^2 and Fisher's exact tests are provided in the table. Significant *p*-values are in **bold**

Species and area/ comparison	*R. palpebrosa*, all	*R. palpebrosa*, Barents Sea[*]	*R. palpebrosa*, West Greenland[*]	*R. megaptera*, all	*R. megaptera*, Barents Sea[*]	*R. megaptera*, West Greenland[*]	*R. moelleri*, Barents Sea[*, †]
between sexes	χ^2 = 0.80, *d .f.* = 2, *p* = 0.68; *p* = 0.67	χ^2 = 2.54, *d .f.* = 2, *p* = 0.28; *p* = 0.29	χ^2 = 0.22, *d .f.* = 2, *p* = 0.91; *p* = 0.91	χ^2 = 0.04, *d .f.* = 2, *p* = 0.99; *p* = 0.99	χ^2 = 0.71, *d .f.* = 2, *p* = 0.69; *p* = 0.69	χ^2 = 0.19, *d .f.* = 2, *p* = 0.92; *p* = 0.92	χ^2 = 0.14, *d .f.* = 2, *p* = 0.95; *p* = 0.95
among ontogenetic groups	χ^2 = 2.03, *d .f.* = 4, *p* = 0.74	χ^2 = 1.43, *d .f.* = 4, *p* = 0.85	χ^2 = 2.87, *d .f.* = 4, *p* = 0.58	χ^2 = 0.28, *d .f.* = 4, *p* = 0.99	χ^2 = 8.91, *d .f.* = 4, *p* = 0.06	χ^2 = 1.90, *d .f.* = 4, *p* = 0.76	χ^2 = 2.14, *d .f.* = 4, *p* = 0.72

[*]General comparison among species and areas: χ^2 = 3.71, *d .f.* = 8, *p* = 0.88;

[†]Only *R. moelleri* from the Barents Sea had their diet modeled.

Supplementary Figure 1. Simulated mixing polygons to check the data fitting to the prey group sources and trophic enrichment factors, as suggested in Smith *et al.*[1]. Consumers (studied specimens; black dots) and average source signatures (white crosses) are shown. Exact values of δ^{13}C and δ^{15}N in studied specimens are in Supplementary Table 1, exact values of sources and their standard deviations are in Table 4, trophic enrichment factors and their standard deviations are in Methods

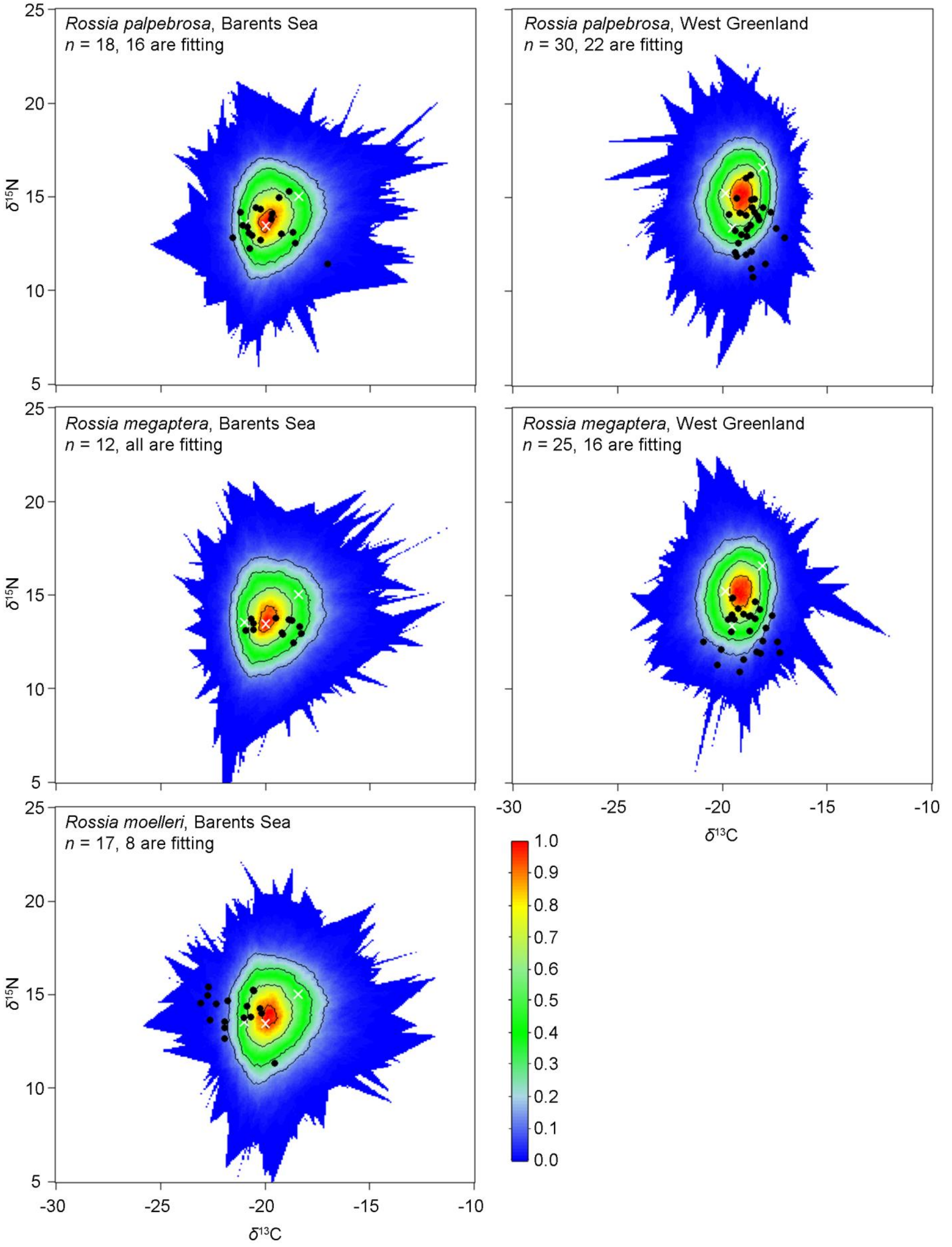

Supplementary Figure 2. Relative contribution of prey to the individual diet in each fitting specimen of the genus *Rossia* predicted by Bayesian mixing model SIMMR 0.4.1

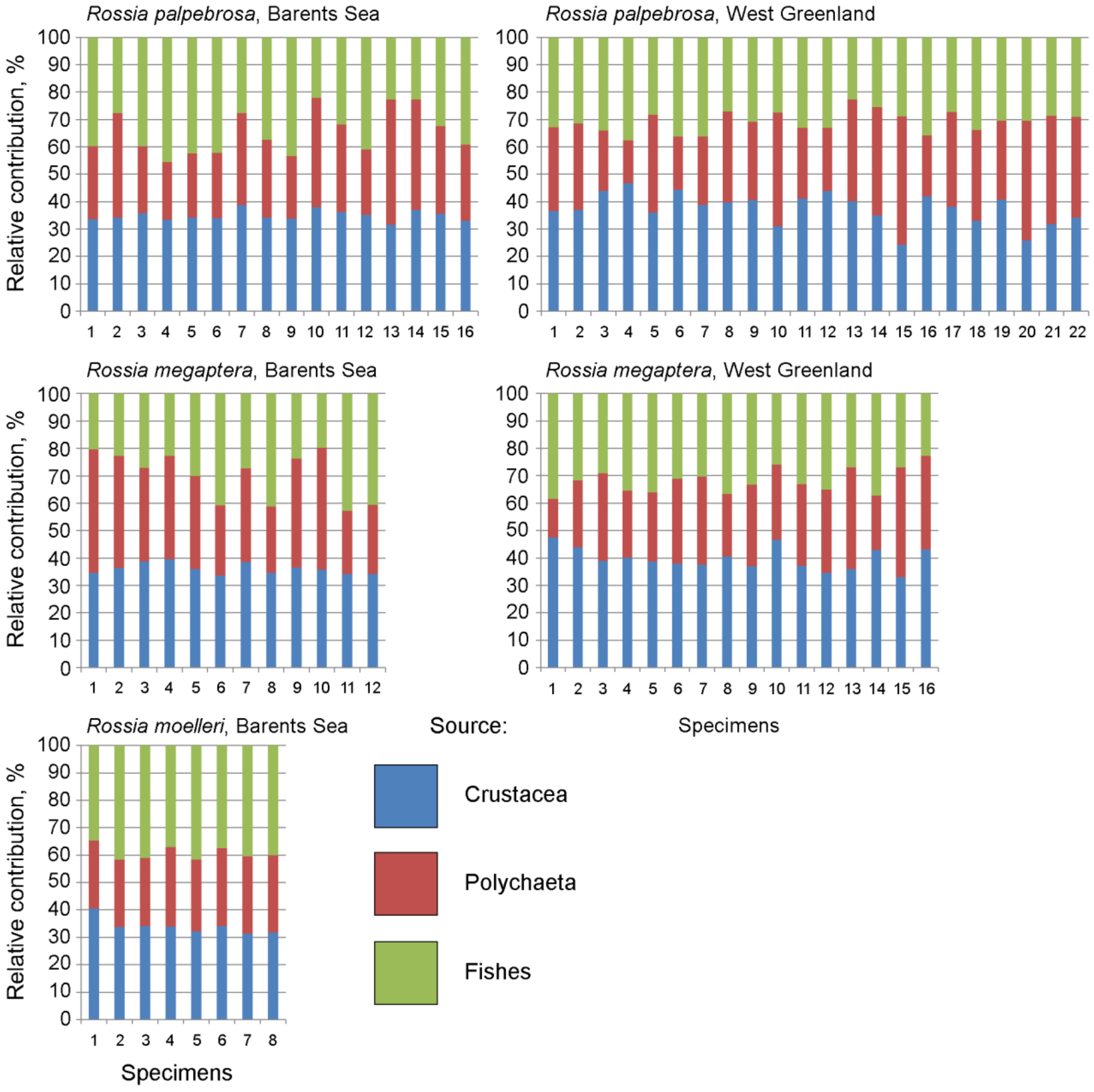

Reference:

1. Smith, J.A., Mazumder, D., Suthers, I.M. & Taylor, M.D. To fit or not to fit: evaluating stable isotope mixing models using simulated mixing polygons. *Methods Ecol. Evol.* **4**, 612–618 (2013).
